# The High Expression of PD-1 Defines A Subpopulation of Tfh Cells Responding to COVID-19 Vaccine in Humans

**DOI:** 10.1093/gpbjnl/qzaf019

**Published:** 2025-03-13

**Authors:** Jingxin Guo, Zhangfan Fu, Yi Zhang, Mengyuan Xu, Jinhang He, Haocheng Zhang, Qiran Zhang, Jieyu Song, Ke Lin, Mingxiang Fan, Zhangyufan He, Guanmin Yuan, Ning Jiang, Huang Huang, Chao Qiu, Jingwen Ai, Wenhong Zhang

**Affiliations:** Department of Infectious Diseases, Shanghai Key Laboratory of Infectious Diseases and Biosafety Emergency Response, National Medical Center for Infectious Diseases, Huashan Hospital, Shanghai Medical College, Fudan University, Shanghai 200040, China; Shanghai Sci-Tech Inno Center for Infection & Immunity, Shanghai 200052, China; Department of Infectious Diseases, Shanghai Key Laboratory of Infectious Diseases and Biosafety Emergency Response, National Medical Center for Infectious Diseases, Huashan Hospital, Shanghai Medical College, Fudan University, Shanghai 200040, China; Department of Infectious Diseases, Shanghai Key Laboratory of Infectious Diseases and Biosafety Emergency Response, National Medical Center for Infectious Diseases, Huashan Hospital, Shanghai Medical College, Fudan University, Shanghai 200040, China; Shanghai Sci-Tech Inno Center for Infection & Immunity, Shanghai 200052, China; Department of Infectious Diseases, Shanghai Key Laboratory of Infectious Diseases and Biosafety Emergency Response, National Medical Center for Infectious Diseases, Huashan Hospital, Shanghai Medical College, Fudan University, Shanghai 200040, China; Department of Infectious Diseases, Shanghai Key Laboratory of Infectious Diseases and Biosafety Emergency Response, National Medical Center for Infectious Diseases, Huashan Hospital, Shanghai Medical College, Fudan University, Shanghai 200040, China; Department of Infectious Diseases, Shanghai Key Laboratory of Infectious Diseases and Biosafety Emergency Response, National Medical Center for Infectious Diseases, Huashan Hospital, Shanghai Medical College, Fudan University, Shanghai 200040, China; Department of Infectious Diseases, Shanghai Key Laboratory of Infectious Diseases and Biosafety Emergency Response, National Medical Center for Infectious Diseases, Huashan Hospital, Shanghai Medical College, Fudan University, Shanghai 200040, China; Department of Infectious Diseases, Shanghai Key Laboratory of Infectious Diseases and Biosafety Emergency Response, National Medical Center for Infectious Diseases, Huashan Hospital, Shanghai Medical College, Fudan University, Shanghai 200040, China; Department of Infectious Diseases, Shanghai Key Laboratory of Infectious Diseases and Biosafety Emergency Response, National Medical Center for Infectious Diseases, Huashan Hospital, Shanghai Medical College, Fudan University, Shanghai 200040, China; Department of Infectious Diseases, Shanghai Key Laboratory of Infectious Diseases and Biosafety Emergency Response, National Medical Center for Infectious Diseases, Huashan Hospital, Shanghai Medical College, Fudan University, Shanghai 200040, China; Department of Infectious Diseases, Shanghai Key Laboratory of Infectious Diseases and Biosafety Emergency Response, National Medical Center for Infectious Diseases, Huashan Hospital, Shanghai Medical College, Fudan University, Shanghai 200040, China; Department of Infectious Diseases, Shanghai Key Laboratory of Infectious Diseases and Biosafety Emergency Response, National Medical Center for Infectious Diseases, Huashan Hospital, Shanghai Medical College, Fudan University, Shanghai 200040, China; Department of Infectious Diseases, Shanghai Key Laboratory of Infectious Diseases and Biosafety Emergency Response, National Medical Center for Infectious Diseases, Huashan Hospital, Shanghai Medical College, Fudan University, Shanghai 200040, China; Department of Infectious Diseases, Shanghai Key Laboratory of Infectious Diseases and Biosafety Emergency Response, National Medical Center for Infectious Diseases, Huashan Hospital, Shanghai Medical College, Fudan University, Shanghai 200040, China; Department of Infectious Diseases, Shanghai Key Laboratory of Infectious Diseases and Biosafety Emergency Response, National Medical Center for Infectious Diseases, Huashan Hospital, Shanghai Medical College, Fudan University, Shanghai 200040, China; Department of Infectious Diseases, Shanghai Key Laboratory of Infectious Diseases and Biosafety Emergency Response, National Medical Center for Infectious Diseases, Huashan Hospital, Shanghai Medical College, Fudan University, Shanghai 200040, China; Shanghai Sci-Tech Inno Center for Infection & Immunity, Shanghai 200052, China; Shanghai Huashen Institute of Microbes and Infections, Shanghai 200052, China

**Keywords:** COVID-19, Booster vaccination, PD-1^high^ cTfh, Virus-induced TCR, Immune persistence

## Abstract

Inactivated coronavirus disease 2019 (COVID-19) vaccines and receptor-binding domain subunit (RBD-subunit) booster vaccination can induce effective humoral immune responses. CD4^+^ T helper cells are essential for helping B cells and antibody responses. However, the response of CD4^+^ T cells to booster vaccination, especially the virus-induced T follicular helper (Tfh) cells, needs to be better characterized. In this study, we investigated this response using single-cell sequencing and flow cytometry. Additionally, we employed a customized algorithm to identify virus-induced T cell receptors (VI-TCRs), enabling further exploration of the activation and persistence of virus-induced CD4^+^ T cell responses. We identified a subset of classic Tfh (cTfh) cells with high expression of PD-1 and IFN-γ. These cells were notably activated following booster vaccination, and their proportion was correlated with antibody titers. Trajectory analysis of activated cTfh cells revealed a subset of virus-induced cTfh cells that might maintain immune responses beyond 90 days post-vaccination. In summary, we identified a group of PD-1^high^ cTfh cells induced by COVID-19 vaccination, which can enhance humoral responses and exhibit the long-term persistence against severe acute respiratory syndrome coronavirus 2 (SARS-CoV-2). We also developed a method for single-cell immune data analysis to understand virus-induced immune responses. Understanding how cTfh cells help antibody production will provide essential insights into the rational design of new vaccine strategies to optimize long-term immunity.

## Graphical abstract

**Figure:**
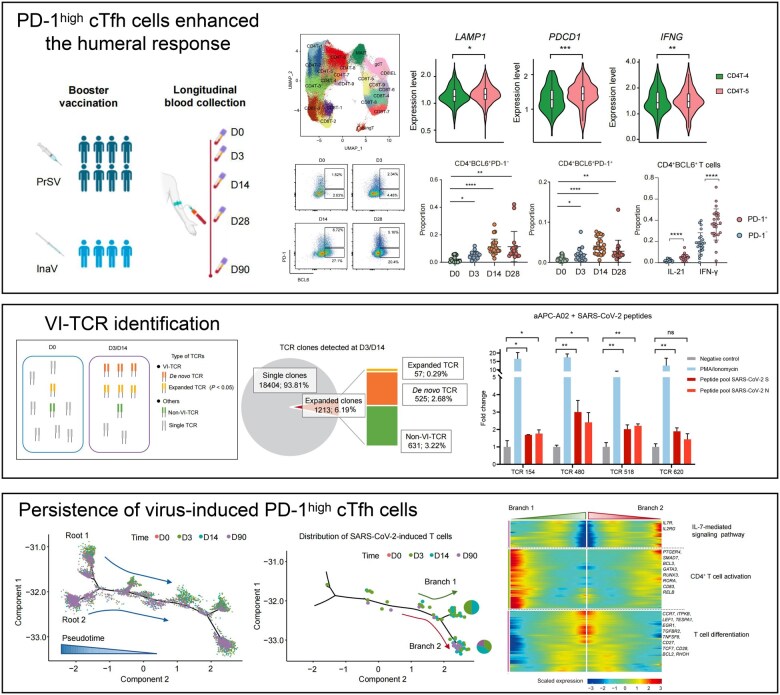

## Introduction

The coronavirus disease 2019 (COVID-19) caused by severe acute respiratory syndrome coronavirus 2 (SARS-CoV-2) is still ongoing. Vaccination is the most cost-effective tool to curtail the spread of COVID-19. To date, various types of COVID-19 vaccines, including inactivated virion, viral vector, protein subunit, messenger RNA (mRNA), and virus-like particle (VLP) vaccines, have been licensed for clinical use [1]. Most of these vaccines induce B cells to generate neutralizing antibodies that protect against SARS-CoV-2 infection [2–6]. However, less attention has been paid to the role of CD4^+^ T helper cells, particularly T follicular helper (Tfh) cells, which are essential for modulating the B cell response. Tfh cells interact with B cell subsets in germinal centers to affect immunoglobulin affinity maturation, B cell differentiation, and the formation of memory B cells [7–9]. Sampling and examining the classic Tfh (cTfh) cells that traffic in the peripheral blood are practical in clinical research. There are different subsets of cTfh cells [7], and alterations in the frequencies and phenotypes of these subsets are associated with disease pathophysiology [10–13]. Whether there are changes in cTfh cells after COVID-19 vaccination remains unknown. Therefore, we characterized the response of cTfh cells to COVID-19 booster vaccination in humans and explored its association with the generation of neutralizing antibodies against SARS-CoV-2.

The lack of a reliable, sensitive, and efficient approach to identify T cell clones that respond specifically to a given antigen has hindered efforts to track the biological processes of virus-specific Tfh cells after vaccination. Single-cell 5′ mRNA and V(D)J sequencing [scRNA/V(D)J-seq] is an emerging technique that enables the measurement of gene expression and antigen–receptor diversity at single-cell resolution as cell lineages undergo dynamic changes. Previous scRNA/V(D)J-seq studies have reconstructed the differentiation of T cells of interest, especially tumor-infiltrating lymphocytes, in cross-sectional samples, which requires experimental alignment of T cell clonotypes and recognition of their responsiveness to specific epitopes [14,15]. By collecting samples at multiple time points, it is possible to track the phenotype alteration and long-term persistence of each vaccine-specific Tfh cell clonotype after vaccination, which is crucial for immune memory.

In this study, we performed scRNA/V(D)J-seq and flow cytometry to investigate virus-specific cellular responses to a COVID-19 booster vaccine. We enrolled participants who received homologous BBIBP-CorV/BBIBP-CorV or heterologous BBIBP-CorV/ZF2001 booster vaccination. Because of the strict public health measures to prevent COVID-19 in China, it is highly likely that the participants’ immune responses to SARS-CoV-2 were due solely to vaccination rather than natural infection. Vaccine-specific T cells are more likely to expand and be detected after a booster dose than initially after a primary vaccination series. In this study, we collected samples after booster vaccination and identified a population of activated cTfh cells that expressed activation markers, particularly PD-1, and functionally produced IL-21 and IFN-γ. The size of this activated cTfh population was correlated with the magnitude of the antibody response. In addition, we employed a customized algorithm for virus-induced T cell receptor (VI-TCR) identification and identified 582 SARS-CoV-2-specific TCRs. By tracking the activation and persistence of VI-TCR cells within the activated cTfh population, we found that these cells regulated antibody production, providing a theoretical basis for developing new vaccines. Two-thirds of the participants experienced breakthrough infection in January 2023, allowing us to validate our approach for identifying vaccine-specific T cells.

## Results

### The dynamics of T cell composition following booster vaccination

The study enrolled 134 volunteers to receive a third dose of a COVID-19 vaccine. All participants had no prior history of SARS-CoV-2 infection and were administered either homologous BBIBP-CorV/BBIBP-CorV or heterologous BBIBP-CorV/ZF2001 booster vaccination. Blood samples were collected at various time points: day 0 (D0), day 3 (D3), day 14 (D14), day 28 (D28), and day 90 (D90). All participants remained SARS-CoV-2 infection-free for at least six months after booster vaccination. When breakthrough infections were reported in January 2023, affected participants were recalled for additional blood collection within one month. To assess the neutralizing antibody titer, a pseudovirus neutralization test (pVNT) was conducted on all plasma samples collected at each time point.

**Figure 1:**
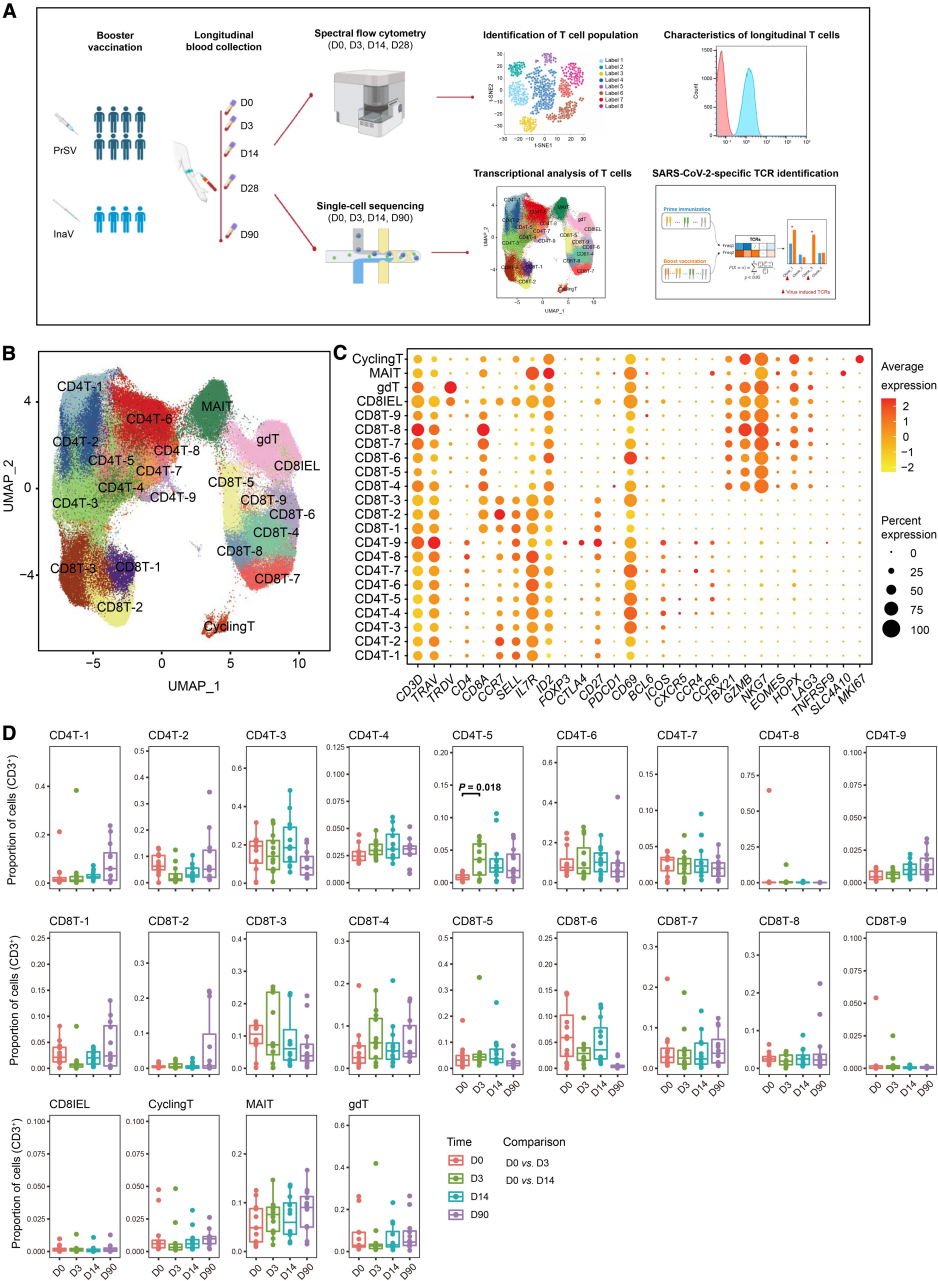
Single-cell profiling of T cell immune dynamics after booster vaccination **A**. Schematic diagram of sample collection and experimental design. All 12 participants received three doses of vaccination, with four receiving an inactivated vaccine (InaV) and eight receiving a recombinant vaccine (PrSV). Blood was collected from each participant on D0, D3, D14, D28, and D90. PBMCs from D0, D3, D14, and D28 were utilized for Cytek flow cytometry, and PBMCs from D0, D3, D14, and D90 were used for scRNA-seq and scV(D)J-seq. Plasma was used to detect SARS-CoV-2 neutralizing antibody titers. **B**. UMAP visualization of T cell populations from all participants and time points. **C**. Heatmap showing the scaled expression of canonical marker genes across all identified T cell clusters. **D**. Proportion variation of each T cell cluster at four time points (D0, D3, D14, and D90) after the third vaccination. Comparisons were performed between the indicated time points (D0 *vs.* D3 and D0 *vs.* D14), with only significant difference being labeled (*P* < 0.05; Mann–Whitney U test). scRNA-seq, single-cell RNA sequencing; scV(D)J-seq, single-cell V(D)J sequencing; PBMC, peripheral blood mononuclear cell; SARS-CoV-2, severe acute respiratory syndrome coronavirus 2; UMAP, uniform manifold approximation and projection; V, variable; D, diversity; J, joining; TCR, T cell receptor; MAIT, mucosal-associated invariant T; D0, day 0.

In the single-cell experiments, peripheral blood mononuclear cells (PBMCs) were collected from 12 participants at D0, D3, D14, and D90 post-booster vaccination and within one month after breakthrough infection. Each time point included two participants from the high-titer homologous booster group (InaV-H), two from the low-titer homologous booster group (InaV-L), four from the high-titer heterologous booster group (PrSV-H), and four from the low-titer heterologous booster group (PrSV-L), providing a comprehensive single-cell profile of the immune response following booster vaccination. We utilized 32-color cytometry (Table S1) and scRNA/V(D)J-seq to explore T cell characteristics associated with antibody responses. After filtering out low-quality cells, we obtained single-cell transcriptomic data for 179,728 T cells and single-cell TCR sequence data for 83,131 T cells. Of these, 64,603 cells had both high-quality gene expression and TCR profiling data (**Figure 1**A).

To characterize the heterogeneity of the entire T cell population, we used a graph-based approach for unsupervised clustering of all sequenced T cells. We visualized the results in a two-dimensional (2D) space using uniform manifold approximation and projection (UMAP) based on the gene expression profiles. In total, we identified 22 distinct clusters representing different cell types, including nine clusters of CD4^+^ T cells (Figure 1B). We manually annotated the cell types based on expression of canonical marker genes [16] (Figure 1C). Three of the CD4^+^ T cell clusters (CD4T-1, CD4T-2, and CD4T-3) constituted naïve/resting CD4^+^ T cells, characterized by the naïve T cell marker genes (*SELL*, *CCR7*, *TCF7*, and *LEF1*) and the resting memory T cell marker genes (*IL7R* and *AQP3*). The cells in cluster CD4T-1 were conventional naïve CD4^+^ T cells, those in cluster CD4T-2 were central memory CD4^+^ T cells with high expression of *SELL*, *CCR7*, and *AQP3*, and those in cluster CD4T-3 were memory cells with high expression of *ID2*. The cells in clusters CD4T-4, CD4T-5, CD4T-6, CD4T-7, and CD4T-8 were separately annotated as classic cTfh cells, activated cTfh cells, Th1/Th17-like cells, Th2 cells, and Th17 cells, respectively. The cells in cluster CD4T-9 were regulatory CD4^+^ T (Treg) cells with high expression of the Treg marker genes *FOXP3*, *CTLA4*, and *IL2RA* (Figure 1C).

### CD4^**+**^ T cells were activated after booster vaccination

**Figure 2:**
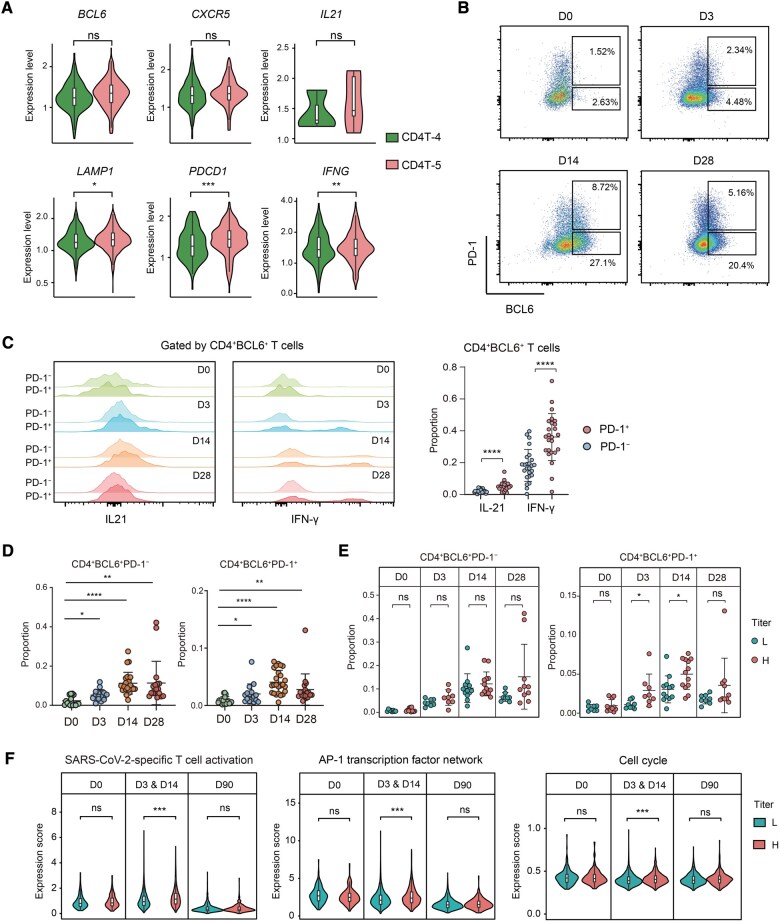
Functional comparison of classic and activated cTfh cells **A.** Violin plots showing the expression of Tfh-relative markers in CD4T-4 and CD4T-5 cell subsets. **B**. Flow cytometric identification of CD4^+^BCL6^+^PD-1^+^ and CD4^+^BCL6^+^PD-1^−^ T cells at D0, D3, D14, and D28 post-booster vaccination. **C**. Left: MFI of IL-21 and IFN-γ within CD4^+^BCL6^+^ T cells at D0 (green), D3 (blue), D14 (orange), and D28 (red). Right: dot plot showing the proportions of IL-21^+^ and IFN-γ^+^ cells in CD4^+^BCL6^+^ T cells collected at D14. Data are represented as mean ± SD. **D**. Dot plots showing the proportions of CD4^+^BCL6^+^PD-1^+^ and CD4^+^BCL6^+^PD-1^−^ T cells over four time points. **E**. Dot plots showing the proportions of CD4^+^BCL6^+^PD-1^+^ and CD4^+^BCL6^+^PD-1^−^ T cells between the H and L groups over four time points. **F**. Violin plots showing the expression scores of three gene expression patterns: SARS-CoV-2-specific T cell activation, AP-1 transcription factor network, and cell cycle between the H and L groups during immunization. In (A and C–F), statistical significance was determined by *t*-test (*, *P* < 0.05; **, *P* < 0.01; ***, *P* < 0.001; ****, *P* < 0.0001; ns, not significant). Tfh, T follicular helper; cTfh, classic Tfh; MFI, mean fluorescence intensity; SD, standard deviation; H, high antibody titer; L, low antibody titer.

To understand the dynamic changes of T lymphocytes after booster vaccination, we compared the proportions of CD4^+^ T cells at the early and late stages (D0 *vs.* D3, D0 *vs.* D14, and D0 *vs.* D90). None of the CD4^+^ T cell clusters changed significantly after booster vaccination, except for cluster CD4T-5, which consisted of activated cTfh cells. The proportion of CD4T-5 was increased at the early stage (D3) after booster immunization (*t*-test, *P* = 0.018) (Figure 1D). Tfh cells are a specialized subset of CD4^+^ T cells required for germinal centers and related B cell responses [7,17,18]. Compared with classic cTfh cells (cluster CD4T-4), activated cTfh cells (cluster CD4T-5) had higher expression of *LAMP1*, *IFNG*, and *PDCD1* (**Figure 2**A). Previous studies have reported that early activation of Tfh cells after vaccination is responsible for humoral immune responses [19,20]. In addition to comparing CD4^+^ T cell clusters at different time points, we compared them between participants who received homologous or heterologous booster vaccination, as well as between participants with high or low antibody titers. The clusters showed similar distributions after homologous and heterologous booster vaccinations; however, the proportion of mucosal-associated invariant T (MAIT) cells at D3 was higher among the participants with high antibody titers than among those with low antibody titers (Figure S1).

### High antibody titers were associated with activated cTfh cells

We conducted flow cytometry to further confirm the characteristics of the cells in clusters CD4T-4 and CD4T-5. The variation of CD4^+^ T cells at D0, D3, D14, and D28 after booster vaccination did not suggest any apparent post-booster regulation (Figures S2 and S3), except for the two clusters of cTfh cells, which exhibited apparent functional differences in both single-cell RNA sequencing (scRNA-seq) and flow cytometry analyses (Figure 2A and B). scRNA-seq results showed that the cells in both clusters expressed *BCL6*; however, *PDCD1* and *IFNG* expression was significantly higher in the activated cTfh cells than in the classic cTfh cells (Figure 2A). The flow cytometry analysis also revealed apparent differences in PD-1 expression between the classic and activated cTfh cells at all four time points from D0 to D28 (Figure 2B). Furthermore, the mean fluorescence intensities of the cytokines IL-21 and IFN-γ were higher in the activated cTfh cells than in the classic cTfh cells from D3 to D28 (Figure 2C, left). The proportions of cells secreting IL-21 and IFN-γ were also higher in the activated cTfh cells than in the classic cTfh cells (*P* < 0.0001 at D14) (Figure 2C, right). The classic and activated cTfh cell subsets both expanded from D3 to D28 (Figure 2D). However, the activated cTfh cell subset was proportionally higher in the participants with high antibody titers than in those with low antibody titers at D3 and D14 (Figure 2E, right). In contrast, there was no significant difference in the classic cTfh cell subset size between the participants with high and low antibody titers (Figure 2E, left). These results suggest that a subset of cTfh cells is activated after booster vaccination and contributes to antibody production. We then sorted CXCR5^+^PD-1^high^ (activated cTfh) and CXCR5^+^PD-1^low^ (classic cTfh) cells and co-cultured them with naïve B cells. Activated cTfh cells helped naïve B cells produce more antibodies at D3 compared to the CXCR5^+^PD-1^low^ group, and the activation level of naïve B cells was higher (Figures S4 and S5). Additionally, the scRNA-seq analysis indicated that genes related to SARS-CoV-2-specific T cell activation, AP-1 transcription factor network, and cell cycle were more highly expressed in the participants with high antibody titers than in those with low antibody titers at D3 and D14 (Figure 2F), indicating that activation of Tfh cells plays a role in the production of SARS-CoV-2-specific antibodies. We further validated the presence of activated cTfh cells in different contexts of SARS-CoV-2 infection. The activated cTfh cells were identified based on the expression of marker genes *LAMP1* and *PDCD1*. Notably, the population of activated cTfh cells was enriched in the abortive infection group of a human SARS-CoV-2 challenge study (Figure S6), and exhibited a negative correlation with the severity of COVID-19 (Figure S7) and the incidence of post-acute sequelae of COVID-19 (Figure S8) in a large cohort of COVID-19 patients followed from initial diagnosis to convalescence (2–3 months later). These findings suggest that activated cTfh cells may respond rapidly upon viral infection, helping to control infection and prevent disease progression. Collectively, we reveal an activated cTfh cell subset, which plays an essential role in enhancing antibody titers following SARS-CoV-2 vaccination, stemming the tide of the progression of COVID-19 disease severity and long COVID.

### Identification of SARS-CoV-2-induced TCR clones

**Figure 3:**
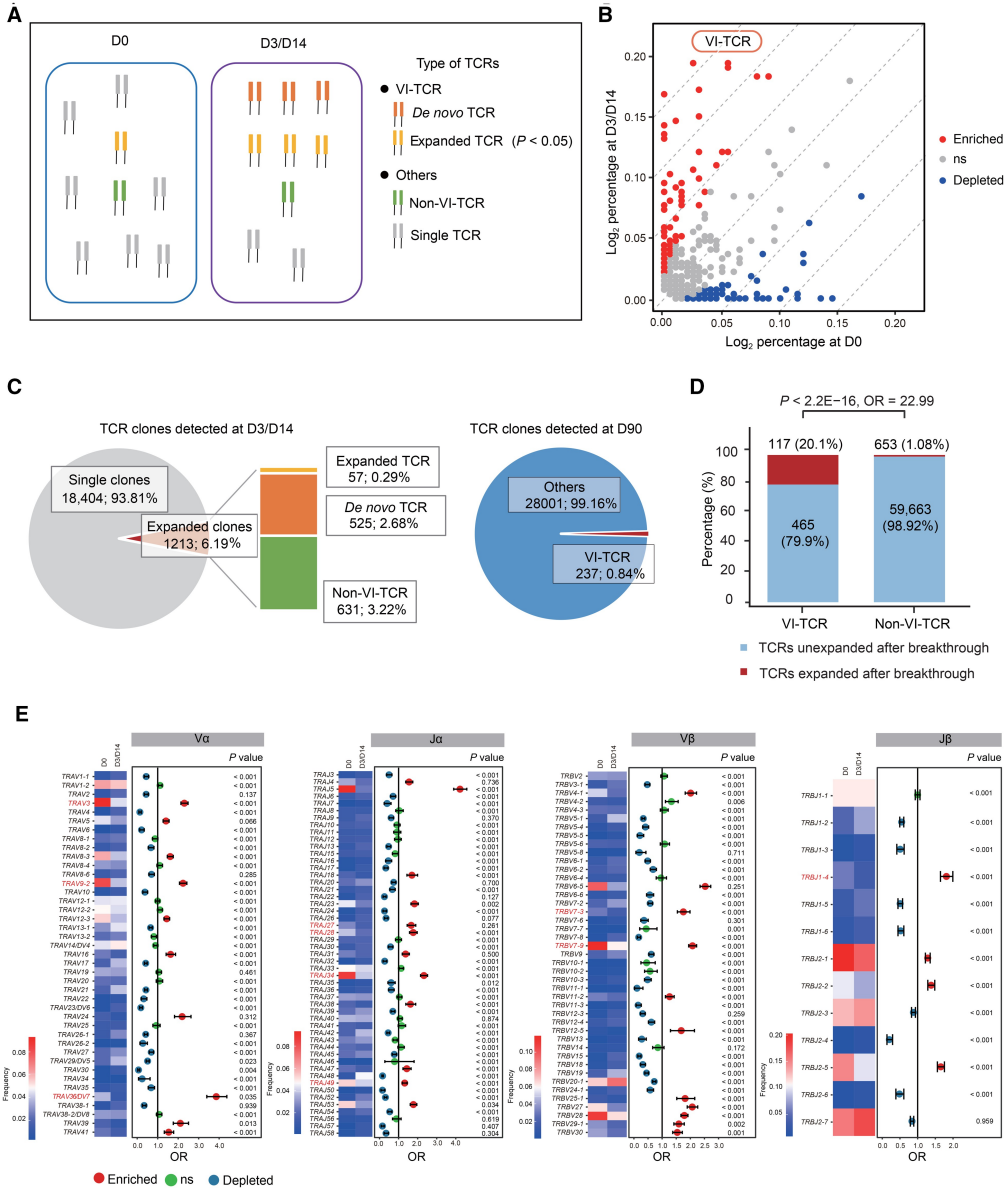
Application of the VI-TCR algorithm to characterize VI-TCRs after booster vaccination **A**. Schematic diagram of the VI-TCR algorithm to characterize VI-TCRs after booster vaccination. Dark orange denotes *de novo* TCR; light orange denotes expanded TCR; green denotes non-VI-TCR; and gray denotes single TCR. *P* < 0.05 (Fisher’s exact test). **B**. Volcano plot showing the enrichment and depletion of TCRs at D3/D14 *vs.* D0. Red dots denote enriched TCRs (OR > 1 and *P* < 0.05), blue dots denote depleted TCRs (OR < 1 and *P* < 0.05), and gray dots denote TCRs without significant variation. **C**. Left: the pie chart shows the distributions of single (gray) and expanded (red) TCR clones at D3/D14 (two time points together), and the stacked plot further shows the distributions of *de novo* TCRs (bright orange), expanded TCRs (dark orange), and non-VI-TCRs (green). Right: the pie chart shows the distributions of VI-TCRs (red) and other TCRs (blue) at D90. **D**. Expansion comparison of VI-TCRs and non-VI-TCRs after breakthrough infection. **E**. Heatmaps combined with forest plots displaying the usage frequencies and enrichment of V and J gene segments of *TRA* and *TRB* in VI-TCR clones at D3/D14 compared to D0. For each gene segment, the heatmap shows the frequencies at D3/D14 *vs.* D0; the forest plot displays the OR for each gene segment, with horizontal lines indicating 95% confidence intervals; statistical significance (*P* < 0.05) is determined by Fisher’s exact test based on the frequency comparison between the two time points. Red dots denote OR > 1 and *P* < 0.05 (enriched at D3/D14) and blue dots denote OR < 1 and *P* < 0.05 (depleted at D3/D14). Genes highlighted in red are those previously reported to be associated with SARS-CoV-2 infection. OR, odds ratio; VI-TCR, virus-induced TCR; non-VI-TCR, non-virus-induced TCR.

From the scRNA/V(D)J-seq dataset, we identified 83,131 T cells with complete TCR α chain (TRA) and TCR β chain (TRB) sequences. After booster vaccination, SARS-CoV-2-specific T cells were activated and underwent clonal expansion, proliferating rapidly to produce many T cells with identical TCR sequences. Although most T cells in our dataset contained unique TCR sequences, we observed varying degrees of clonal overlap between D3/D14 and D90. Based on these results, we developed a customized algorithm to identify SARS-CoV-2-induced TCRs based on clone frequency (**Figure 3**A). Comparison of the T cell repertoires between D3/D14 and D0 using this algorithm identified 582 VI-TCRs (*P* < 0.05, Fisher’s exact test) (Figure 3B). These VI-TCRs were classified into two groups: *de novo* TCRs (uniquely detected at D3/D14) and expanded TCRs (significantly increased at D3/D14). Approximately 2.97% of the T cells harbored VI-TCRs at the early stage after booster vaccination. Furthermore, 2.68% of the VI-TCRs detected at D3/D14 were *de novo* SARS-CoV-2-induced TCRs. At D90, 0.84% of the detected TCR clones were VI-TCRs (Figure 3C), and these persistent TCRs could be detected in both the homologous and heterologous booster groups. Notably, 117 of the 582 VI-TCRs were significantly expanded after breakthrough infection [breakthrough *vs*. D90: odds ratio (OR) = 22.99, *P* < 2.2E−16], which validated the accuracy of the VI-TCR algorithm (Figure 3D). We further verified the specificity of these VI-TCRs for the spike (S) and nucleocapsid (N) antigens of SARS-CoV-2 and found that all selected VI-TCRs bound to the S and N proteins (Figure S9; Tables S2 and S3).

TCRs are generated by the rearrangement of variable (V), diversity (D), and joining (J) gene segments for *TRB* and V and J gene segments for *TRA*. To further characterize the VI-TCR clones, we explored the usage bias of the V and J gene segments in the TCR clones detected at D3/D14 compared with those detected at D0. Comparison of every V and J gene segments revealed that the frequencies of some V and J gene segments in the α/β chain significantly changed at D3/D14 compared to those at D0 (*P* < 0.05, Fisher’s exact test) (Figure 3E). Among the TCR clones detected at D3/D14, the most frequently used gene segments were *TRAV36/DV7*, *TRAJ5*, *TRBV6-5*, and *TRBJ1-4*. Consistent with previous studies [21,22], we also observed increased frequencies of some V gene segments, including *TRAV3*, *TRAV9-2*, *TRAV36/DV7*, *TRBV7-3*, and *TRBV7-9*. These results indicate that the preferential usage of TCR gene segments is significantly different between virus-specific TCR clones and the overall TCR repertoire, and the increased virus-specific TCR clones are correlated with SARS-CoV-2 infection.

### The transcriptome of virus-specific CD4^+^ T cells

**Figure 4:**
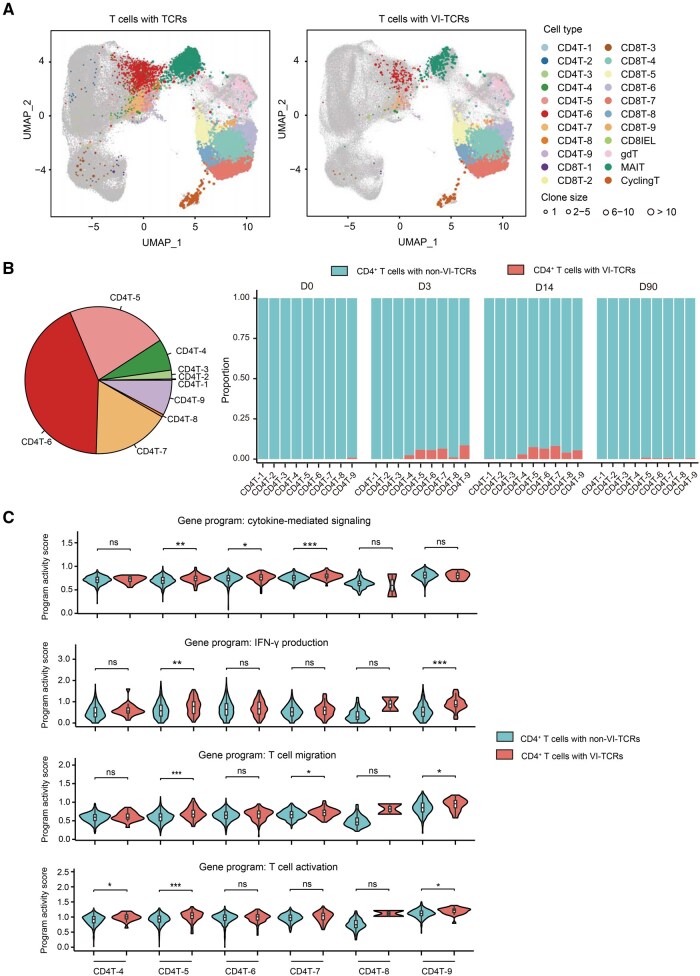
Characterization of CD4**^+^** T cells with VI-TCRs during immunization **A**. UMAP showing CD4^+^ T cells with VI-TCRs. Clusters are denoted by color and labeled with inferred cell types. **B**. Left: pie chart showing the proportion of CD4^+^ VI-TCR cell subsets and labeled with cell types. Right: stacked plot showing the proportion of non-VI-TCR cells and VI-TCR cells at each time point. **C**. Violin plots showing the program activity scores of gene expression patterns in non-VI-TCR and VI-TCR cells within each inferred cell subset.

To gain more insight into the virus-specific T cells, we evaluated changes in gene expression and TCR repertoire of the major subsets of CD4^+^ T cells. Using paired single-cell TCR sequencing (scTCR-seq), we assessed TCR clonal expansion at different time points. Of the 64,603 cells for which both gene expression and TCR profiling data were obtained, 6662 T cells were identified as carrying VI-TCRs (**Figure 4**A). We found that virus-specific clones expanded after booster vaccination, with expansion mainly observed in T cells that produce cytokines and exhibit cytotoxicity (Figure 4B, Figure S10). Comparison of differentially expressed genes between virus-specific T cells and non-virus-induced T cells within each cluster at D3/D14 revealed that genes related to T cell activation were upregulated in the virus-specific expanded T cells within clusters CD4T-4, CD4T-5, and CD4T-9 (Figure 4C). In addition, genes involved in cytokine-mediated signaling were upregulated in the virus-specific expanded T cells within clusters CD4T-5, CD4T-6, and CD4T-7 (Figure 4C), indicating the activation of immune-activating and pro-inflammatory responses in these subsets after booster vaccination. Furthermore, genes related to IFN-γ production were upregulated in the virus-specific expanded T cells within clusters CD4T-5 and CD4T-9 (Figure 4C, Figure S11). IFN-γ has been reported to play a role in the clearance of various viral infections [23], and the frequency of IFN-γ-producing T cells is widely used as a parameter to assess vaccine-induced responses [24]. Collectively, these findings suggest that cells in cluster CD4T-5 may play an important role in vaccine-induced responses.

### Specific activation and long-term persistence of activated cTfh cells

**Figure 5:**
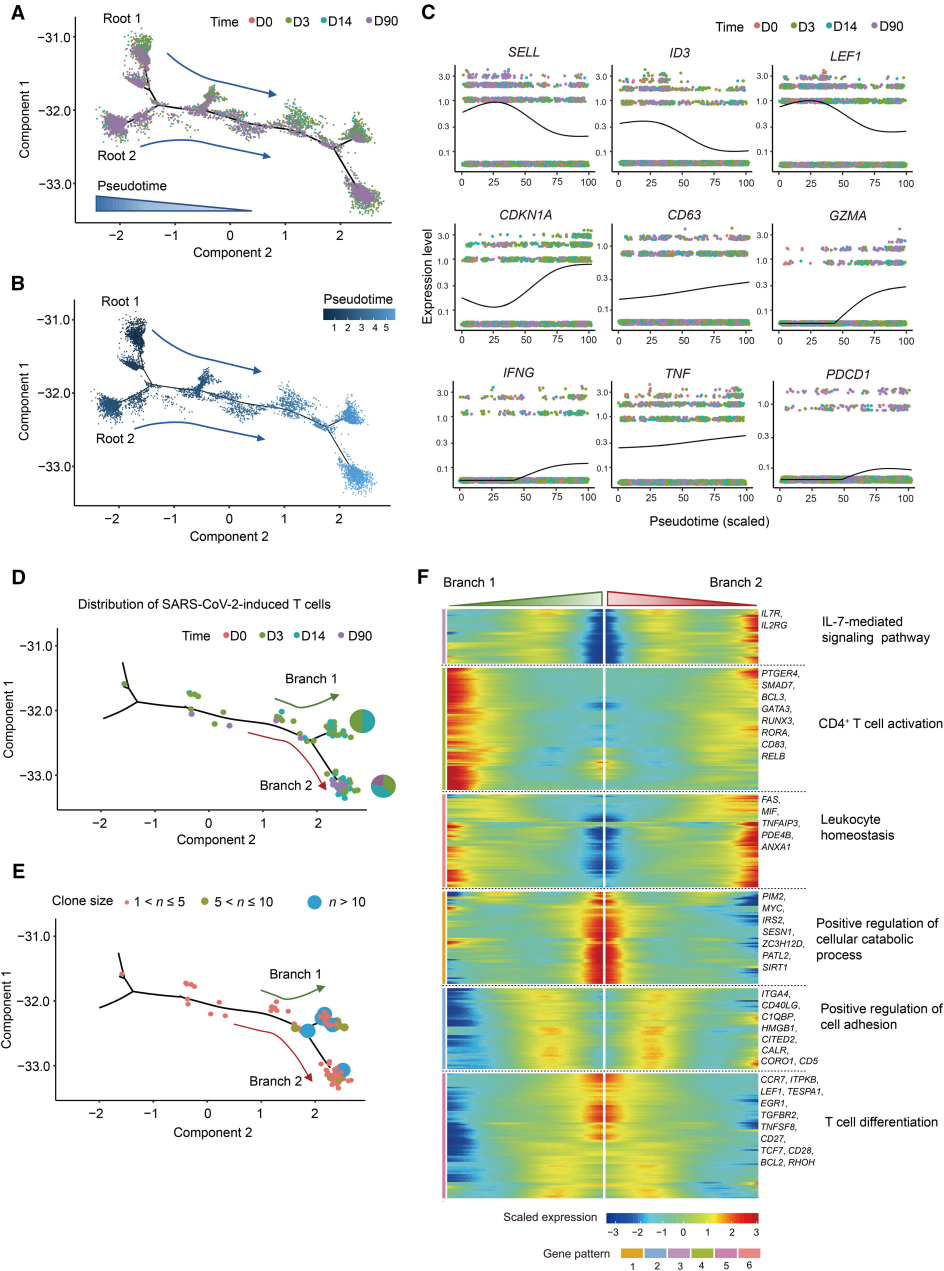
Trajectory analysis on the phenotype shifts of activated cTfh cells over time **A**. Pseudotime trajectories of activated cTfh cells, color-coded by activated cTfh cell phenotypes at D0, D3, D14, and D90 post-booster vaccination. **B**. Pseudotime trajectories of activated cTfh cells, color-coded by pseudotime. **C**. Gene expression dynamics along the activated cTfh cells lineage. The expression of nine genes shows the dynamic transcriptomic changes in activated cTfh at four time points. **D**. and **E**. Pseudotime trajectories of activated cTfh cells with VI-TCRs, color-coded by time point (D) and clone size (E). Green and red arrows indicate branch 1 and branch 2, respectively. **F**. Gene expression proﬁling along the trajectories comparing branch 1 and branch 2.

During adaptive immune responses, Tfh cells play a crucial role in B cell differentiation [25,26]. Virus-specific activated cTfh cells were observed in human blood after exposure to an adenoviral vector, and these cells were correlated with anamnestic IgG responses [27]. We found that cell–cell interactions between Tfh cells and B cells were more robust in individuals with high antibody titers than in those with low antibody titers (Figure S12) [16]. To gain insights into activated cTfh cells, we analyzed their dynamic transcriptomic changes after COVID-19 booster vaccination at four time points (**Figure 5**A and B, Figure S13A). We then modeled the gene expression pattern along the activated cTfh cell lineage (Figure 5C). The expression of marker genes of resting and immature cells, including *LEF1*, *SELL*, and *ID3*, declined over pseudotime following booster vaccination, whereas the expression of genes involved in cytotoxic function (*GZMA*), activation-linked co-stimulation (*CD63*), and T cell activation (*CDKN1A* and *PDCD1*) increased at later time points. The expression of inflammatory factors such as *IFNG* and *TNF* also increased at the end of the pseudotime trajectory.

We next investigated the dynamic changes of activated cTfh cells with VI-TCRs, which hereafter referred to as virus-specific activated cTfh cells. In general, virus-specific activated cTfh cells were activated early in response to booster vaccination and later differentiated into two branches, which we termed branch 1 and branch 2 (Figure 5D and E, Figure S13B). Virus-specific activated cTfh cells from D90 were specifically distributed along branch 2, whereas those in branch 1 were from D3/D14 (Figure S13C and D). Unsupervised clustering analysis divided the genes expressed in the virus-specific activated cTfh cells into six patterns. Dynamic expression analysis showed that genes expressed late in branch 1 were involved in T cell activation, suggesting that the cells in branch 1 are early activated cells (Figure 5F). In contrast, genes expressed late in branch 2 were related to the IL-7-mediated signaling pathway (Figure 5F, Figure S13E). IL-7 is essential for maintaining T cell memory and achieving homeostasis [28], suggesting that the cells in branch 2 may be a group of activated cTfh cells capable of responding to the third vaccination dose. Overall, gene expression profiling along the pseudotime trajectories confirmed that activated cTfh cells were expanded after booster vaccination and that this cell subset maintained cellular immunity with persistence responses after 90 days.

## Discussion

Individuals who receive full primary and booster doses of COVID-19 vaccines have a reduced risk of developing severe COVID-19 [29–32]. It is well established that neutralizing antibodies acquired through vaccination or natural infection provide immunity to SARS-CoV-2, but the levels of these antibodies decay over time [33]. Virus-specific memory T cells may curtail viral infections and protect against disease; however, it remains unclear whether COVID-19 vaccines induce memory T cells capable of conferring long-term immunity to SARS-CoV-2. Here, we performed a comprehensive single-cell analysis of T cell dynamics following a booster dose of either the RBD-subunit vaccine (ZF2001) or the inactivated vaccine (BBIBP-CorV) in individuals who were previously primed with two doses of COVID-19 vaccines.

In this study, we found a subset of activated cTfh cells within the CD4^+^ T cell population that were activated in response to booster vaccination. To further validate the protective role of activated cTfh cells against SARS-CoV-2 infection, we conducted a joint analysis with publicly available datasets. We selected Tfh cells marked by *BCL6* and *CXCR5*, reclustered them, and identified a subcluster highly expressing *PDCD1* and *LAMP1* as activated cTfh cells. We found the activated cTfh cells across multiple datasets and observed a negative correlation between its frequency and the severity of COVID-19 or the incidence of long COVID. Thus, we deciphered an activated cTfh cell subset marked by *PDCD1* and *LAMP1* that plays a beneficial role in the immune response to SARS-CoV-2 infection.

Tfh cells play an important role in antibody affinity maturation and the development of long-lived immune memory [8,18]. In human blood, cTfh cells (CXCR5^+^CD45RA^−^CD4^+^) are heterogeneous and can be classified into cTfh_CM_ (CCR7^high^PD-1^low^), cTfh_EM_ (CCR7^low^PD-1^high^), cTfh1 (CXCR3^+^CCR6^−^), cTfh2 (CXCR3^−^CCR6^−^), and cTfh17 (CXCR3^−^CCR6^+^) subsets [34]. In the context of annual influenza vaccination, a population of activated cTfh cells (CXCR5^+^ICOS^+^Ki67^+^PD-1^+^) is maintained in the blood, and these cells were recalled and expanded by D7 after flu vaccination [35,36]. In our study, activated cTfh cells strongly expressed *IFNG* and *PDCD1*. The activation of these cTfh cells was highly correlated with antibody titers at the early stage after booster vaccination. Once activated, these cells were re-circulated regularly through blood at later stages after vaccination. Additionally, we found that *ICAM* was upregulated in activated cTfh cells after booster vaccination, facilitating the functions of activated cTfh cells on B cells, which vaccine adjuvants might target to boost an auxiliary immune response. These findings suggest that developing adjuvants and immunization methods that enhance the humoral immune response by activating virus-specific activated cTfh cells may be a promising approach for developing next-generation vaccines.

Long-term memory of the T cell response and virus-specific TCRs were recognized even 90 days after booster vaccination. We applied the VI-TCR algorithm to identify virus-specific TCR clones. Of the 582 SARS-Cov-2-specific TCR clones identified by the VI-TCR algorithm, 117 significantly expanded after breakthrough infection, showing that the VI-TCR algorithm is reliable and sensitive for identifying virus-specific TCRs. Furthermore, we profiled the distribution of cells with virus-specific TCRs along the trajectory of activated cTfh cells. Most of the virus-specific activated cTfh cells were activated after booster vaccination, and a subset of these differentiated into a memory state, indicating the existence of long-lasting memory-activated cTfh cells in the blood. For example, CD4^+^ T cells with a VI-TCR clone (TRAV8-2: CAVKNPGGSQGNLIF, TRAJ42; TRBV7-9: CASSLTGDYGYTF, TRBJ1-2) (Table S4) from D3 were distributed on branch 1 and highly expressed CD4^+^ T cell activation genes, but cells with the same clone from D90 were distributed on branch 2 and highly expressed IL-7-mediated pathway genes. CD4^+^ T cells with another VI-TCR clone (TRAV10: CVVSVSGSARQLTF, TRAJ22; TRBV7-2: CASSSGGGGGYTF, TRBJ1-2) (Table S4) from D3 highly expressed CD4^+^ T cell activation genes and shifted to a memory state at D90 (Figure S13D). The VI-TCR algorithm is thus a reliable method for identifying virus-specific induced TCR clones and characterizing dynamic changes of virus-specific T cell responses based on single-cell immune data analysis. Indeed, the VI-TCR algorithm is based on a study comparing longitudinal samples from the same vaccine of TCR clone expansion over time, which is dependent on biological transformation. The VI-TCR algorithm evaluated the differences based on clone frequency and the significance of TCR clones between two conditions through the Fisher’s exact test. However, the Fisher’s exact test may be limited by the sample size and will become unwieldy when the number of possible tables grows exponentially. Thus, we recommend that users combine the VI-TCR algorithm with GLIPH2 (http://50.255.35.37:8080/project) to identify VI-TCR clones and compare their similarity to published clones.

Although CD8^+^ T cells did not exhibit consistent temporal dynamics in our study, they have been shown to play a pivotal role in T cell responses to AZD1222/ChAdOx1 nCoV-19 vaccination and mRNA vaccination [37,38]. We observed higher levels of SARS-CoV-2-specific CD4^+^ T cells in participants with higher antibody titers. This finding aligns with a previous study reporting that older adults with low levels of vaccine-induced CD4^+^ T cells also had low IgG levels [39]. We found that CD4^+^ Tfh cells, dendritic cells, and MAIT cells were correlated with antibody titers after booster vaccination. In a previous study, we found that high antibody titers were associated with active antigen presentation processes by dendritic cells and Tfh cells. In addition, the function of MAIT cells, which includes boosting early immune responses, aiding in defense against viruses, activating adenovirus vector vaccine immunogenicity [40], and altering cell functions, could further contribute to disease severity [41], indicating that expansion of MAIT cells might be a specific response to COVID-19 vaccination.

Our study has several limitations. The limited sample size was insufficient to perform a validation study of the booster immune response effect. Profiling of immune cell populations by scRNA-seq has high resolution (in terms of cell phenotypes and functional characteristics) but a restricted span (in terms of immune repertoire complexity). Thus, although the VI-TCR algorithm improves the sensitivity of virus-specific TCR detection, its accuracy remains dependent on the throughput of scRNA/V(D)J-seq. Additionally, the function of cTfh cells with high PD-1 expression after COVID-19 vaccination remains to be explored *in vivo*. Nevertheless, we successfully performed a parallel investigation of cellular functional dynamics and TCR sequence identity after COVID-19 booster vaccination. Finally, our samples were collected from PBMCs, but most of the Tfh cells exist in the lymph nodes. Further studies are therefore warranted.

## Conclusion

Our study revealed the dynamic changes of CD4^+^ T cells over 90 days following COVID-19 booster vaccination. The levels of activated cTfh cells with high IFN-γ and PD-1 expression were correlated with antibody titers, suggesting that these cells are essential for facilitating antibody production. The VI-TCR algorithm for single-cell immune data analysis provides a new method for understanding the dynamic changes of virus-specific cells. By tracing the differentiation of T cells with virus-specific TCRs identified by the VI-TCR algorithm, we deciphered the activation trajectory and long-lasting immune response of virus-specific activated cTfh cells after COVID-19 booster vaccination. Furthermore, cell–cell interaction analysis revealed enhanced interactions between virus-specific activated cTfh cells and expanded B cells after booster vaccination, suggesting an avenue for the rational design of new vaccine strategies to optimize long-lasting immunity.

## Materials and methods

### Participant enrollment and sample collection

We initiated a prospective cohort study (registered as NCT05095298; Table S5) to assess the immunogenicity of a booster dose of either ZF2001 (heterologous BBIBP-CorV/ZF2001) or BBIBP-CorV (homologous BBIBP-CorV/BBIBP-CorV) following two initial doses of BBIBP-CorV. The study involved 71 and 63 participants in the ZF2001 and BBIBP-CorV booster groups, respectively. Neutralizing antibody levels were measured at D0, D3, D14, D28, and D90 post-booster vaccination [2,20] with additional blood samples collected within one month after breakthrough infection. At each time point, samples were obtained from two participants in the homologous booster high-titer group (InaV-H), two in the homologous booster low-titer group (InaV-L), four in the heterologous booster high-titer group (PrSV-H), and four in the heterologous booster low-titer group (PrSV-L).

### Single-cell collection, sorting, library preparation, and sequencing

PBMCs were extracted using Histopaque-1077 solution (Catalog No. 10771, Sigma-Aldrich, St. Louis, MO), following the manufacturer’s guidelines. Freshly drawn peripheral blood (4 ml) collected in EDTA tubes was carefully layered onto Histopaque-1077 and centrifuged. The PBMCs, which settled at the interface between plasma and Histopaque-1077, were then transferred to a fresh tube. Erythrocytes were lysed using a red blood cell lysis buffer, followed by two washes with a sorting buffer [phosphate-buffered saline (PBS) with 2% fetal bovine serum (FBS; Catalog No. A5669701, Thermo Fisher Scientific, Waltham, MA)]. The resulting cell pellets were suspended in sorting buffer and filtered through a 40-µm Flowmi Cell Strainer (Catalog No. KKE3.1, Carl Roth, Karlsruhe, Germany).

Cryopreserved PBMCs were thawed following the protocol outlined in the User Guide CG00039_Demonstrated. The cells were rapidly thawed in a 37°C water bath and washed in warm Roswell Park Memorial Institute (RPMI) medium (Catalog No. 11875119, Gibco, Waltham, MA) containing 10% FBS. After resuspension in sorting buffer, the cells were filtered through a 40-µm strainer. The single-cell suspension was stained with 7-aminoactinomycin D (7AAD) for viability assessment and sorted using a FACSMelody cell sorter (BD, Franklin Lakes, NJ).

Cell viability, determined using a Countstar Automated Cell Counter (Countstar Rigel S2, Alit Life Science, Shanghai, China), consistently exceeded 90%, and the cell concentration was adjusted to the range of 500–1200 cells/µl. For single-cell sequencing, 18,000 viable PBMCs were loaded per well using the 10X Chromium Next GEM Single Cell 5′ Reagent Kit v2 (Catalog No. 1000263, 10X Chromium, Pleasanton, CA), following the manufacturer’s standard operating procedures. The resulting libraries were sequenced on an Illumina NovaSeq 6000 platform (Catalog No. 20028401, Illumina, San Diego, CA), generating 150-bp paired-end reads.

### Processing of scRNA-seq data and quality control

Cell Ranger (v5.0) was utilized to process the sequencing data, which included filtering out low-quality reads, aligning the reads to the human reference genome (GRCh38), assigning unique cell barcodes, and constructing unique molecular identifier (UMI) matrices. The resulting gene expression data were subsequently analyzed using R software (v3.6.1) in conjunction with the Seurat package (v3.2.0). All sample data were consolidated into a single Seurat object through the merge function. Cells were excluded from the analysis if they exhibited fewer than 200 detected genes, had less than 500 UMI counts, or contained more than 10% mitochondrial UMI counts. The SCTransform function was then applied to normalize the data and identify highly variable genes (HVGs) across the single-cell gene expression dataset. Subsequently, HVGs were curated by excluding mitochondrial genes, genes induced by dissociation, and human leukocyte antigen (HLA) genes to refine the dataset for further analysis. The SCTransform function was also employed to adjust for the variance introduced by the proportion of mitochondrial gene counts, using the parameter “vars.to.regress = percent.mt” to account for this factor in the data normalization process.

### Integrated analysis of single-cell datasets

We performed an integrated scRNA-seq analysis across five time points: D0 (pre-vaccination), D3, D14, and D90 post-vaccination, and the breakthrough time point (January 2023). To adjust for variations introduced by different batches, we implemented the Seurat alignment technique for data integration. This method acknowledges that confounding factors may exert inconsistent effects across the cellular landscape of a dataset and is adept at capturing broad transcriptional variations between datasets. Seurat’s alignment leverages an adaptation of canonical correlation analysis alongside mutual nearest neighbors to detect linear relationships among features and to uncover common correlation patterns spanning multiple datasets. In our approach, we pinpointed variable genes within each dataset, taking into account the pronounced link between gene variability and mean expression levels, thereby normalizing this relationship to ensure a more accurate integration of datasets. We took the union of HVGs from each dataset and performed a correlation analysis to determine the common sources of variation between the five time points. We then aligned the subspaces based on the first 25 canonical correlation vectors, which generated a new dimensionality reduction that was used for further analysis. The resulting 179,728 cells were divided into 22 clusters (Figure 1B). We then separated the cells by treatment to initially examine the transcriptional profile at different states (Figure 1B).

### Dimensionality reduction, unsupervised clustering, and cell-type annotation

Dimensionality reduction and unsupervised clustering were performed using the Seurat’s standard analytical pipeline. Graph-based clustering was applied to the principal component analysis (PCA)-reduced dataset, utilizing the Louvain method following the construction of a shared nearest neighbor graph. The resulting cellular populations were then visualized on a 2D UMAP plot, providing a clear and intuitive representation of the data.

### Differential expression analysis

To identify genes that are differentially expressed between distinct clusters, we employed the FindMarkers function in Seurat to conduct a rigorous differential gene expression analysis. Only genes with an adjusted *P* value below 0.05 were deemed to exhibit significant differential expression, ensuring a robust statistical basis for our findings.

### TCR analysis

We utilized Cell Ranger (v5.0) for the alignment of scTCR-seq reads to the human reference genome (GRCh38), facilitating the assembly of TCR sequences. The preliminary TCR sequences were subjected to a rigorous filtering protocol to retain only high-confidence, full-length, and productive sequences with unambiguous cell barcodes and chain types. For each cell, the α–β chain pair with the highest UMI count was designated to represent its unique TCR signature. Clonal cells, characterized by identical TCR pairs, were identified, indicating a shared lineage. To statistically assess the expansion of specific V–J gene pairs at D3/D14 relative to D0, we applied the Fisher’s exact test. Comparison of group differences was performed using the Mann–Whitney U test for continuous variables or Fisher’s exact test for categorical variables, with statistical significance defined as *P* < 0.05.

### VI-TCR identification

TCRs are generated by the rearrangement of V, D, and J gene segments for *TRB* and V and J gene segments for *TRA*. We performed a customized algorithm for VI-TCR identification to explore the usage bias of V and J gene segments in participants. VI-TCR clones were defined based on the following criteria: (1) detected at D3/D14; (2) clone size > 2; and (3) significantly enriched at D3/D14 compared to D0 (Fisher’s exact test, *P* < 0.05). All statistical analyses were performed using the R software.

### Trajectory inference

We employed Monocle 2 (v2.14.0) to investigate the transcriptional dynamics and functional progression within CD4T-5 cell clusters, as shown in Figure 5A. The raw count data extracted from the Seurat framework were transformed into a Monocle CellDataSet object using the importCDS function. Genes exhibiting differential expression across various subpopulations were designated as the ordering genes to guide the trajectory analysis.

A minimum spanning tree was constructed using the reduceDimensions function, which employs the DDRTree reduction method for accurate representation of cellular progression. Considering the inherent ambiguity in the assignment of pseudotime direction, we designated cells identified at the initial time point (D0) as the entry point of the trajectory, thereby establishing a reference for tracking cellular development and state transitions.

### Cell–cell interaction analysis

We applied CellphoneDB (v2.0), a database of known receptor–ligand pairs, to assess cell–cell communication in our dataset. At D3/D14 and D90, interactions between virus-induced expanded CD4T-5 cells and B cells (both clonally expanded and non-expanded) were compared separately between the high and low antibody titer groups. Interactions were then trimmed based on significant sites with *P* < 0.05.

### TCR- and HLA-specific cell line development

#### Cell culture and cell lines

The J76-NFATRE-luc cells were donated by Dr. Huang Huang. T cells with various TCRs were generated by lentiviral transduction into J76-NFATRE-luc cells. The COS-7 line, sourced from the American Type Culture Collection (ATCC), was cultured under standard conditions. Artificial antigen presenting cells (aAPCs) were developed by transducing CD80, HLA-DM, and diverse HLA alleles into COS-7 cells.

#### Lentiviral TCR transduction

Gene fragments encoding TRA, a P2A linker, and TRB were synthesized by GENEWIZ (Suzhou, China) and cloned into the multiple cloning site (MCS) of the pHR-SFFV-IRES-EGFP(HIG) vector. HEK-293T cells (Catalog No. CRL-3216, ATCC, Manassas, VA) were seeded at a density of 2.4 × 10^6^ cells per 10-cm dish and cultured for 12 h before transfection. Lentiviral particles were produced by co-transfecting HEK-293T cells with 10 μg of the transfer vector, 8 μg of the pMD2.G envelope vector, 4 μg of the psPAX2 packaging vector, and 54 μl of polyethyleneimine (PEI; Catalog No. 40816ES02, YEASEN, Shanghai, China). The medium was refreshed 6 h after transfection, and the viral supernatant was harvested 48 h later. This supernatant was then passed through a 0.45-μm SFCA Syringe Filter (Catalog No. 431219, Corning, Corning, NY) and concentrated by centrifugation. The concentrated lentiviral particles were used to transduce J76-NFATRE-luc cells in the presence of 6 μg/ml polybrene (Catalog No. TR-1003-G, Sigma-Aldrich). TCR expression levels were determined via flow cytometry 48 h post-transduction, and cells expressing TCRs were selected for subsequent epitope screening.

#### Lentiviral HLA transduction

Gene fragments encoding CD80, HLA-DM, and the proteins of diverse HLA alleles were synthesized by GENEWIZ and cloned into the MCS of the pHR-SFFV-IRES-EGFP(HIG) vector. HEK-293T cells were seeded at a density of 2.4 × 10^6^ cells per 10-cm dish and cultured for 12 h before transfection. Lentiviral particles were produced by co-transfecting HEK-293T cells with 10 μg of the transfer vector, 8 μg of the pMD2.G envelope vector, 4 μg of the psPAX2 packaging vector, and 54 μl of PEI (Catalog No. 40816ES02, YEASEN). The medium was refreshed 6 h after transfection, and the viral supernatant was harvested 48 h later. This supernatant was then passed through a 0.45-μm SFCA Syringe Filter (Catalog No. 431219, Corning) and concentrated by centrifugation. The concentrated lentiviral particles were used to transduce COS-7 cells in the presence of 6 μg/ml polybrene (Catalog No. TR-1003-G, Sigma-Aldrich). HLA expression levels were determined via flow cytometry 48 h post-transduction, and HLA-expressing cells were selected for use as aAPCs for subsequent assay.

### SARS-CoV-2 epitope screening

For S and N epitope stimulation, 50 μl of aAPCs at 2 × 10^6^ cells/ml were incubated with S and N epitope pools (detailed in Table S2) at 37°C for 12 h. Subsequently, TCR-transduced J76-NFATRE-luc cells, also at 2 × 10^6^ cells/ml, were added and co-cultured for 6 h in a 96-well plate. Luciferase activity was measured using the Nano-Glo Assay (Catalog No. N1120, Promega, Madison, WI), and fold induction was calculated relative to unstimulated controls. For peptide stimulation, TCR-transduced J76-NFATRE-luc cells were co-cultured with aAPCs in the presence of individual peptides (1 μg/ml; detailed in Table S2). After 6 h of co-culture, luciferase activity was measured, and fold induction was determined by comparison with non-TCR groups.

### Co-culture of PD-1^high^ and PD-1^low^ Tfh cells with naïve B cells

In the co-culture of Tfh cells with varying PD-1 expression levels and B cells, the process began with the isolation and sorting of Tfh cells into PD-1^high^ and PD-1^low^ populations using flow cytometry (FACSAria III, BD). B cells were then isolated from PBMCs and purified for the co-culture. The sorted Tfh cells were mixed with B cells at a 1:5 ratio in a 96-well plate containing RPMI medium (Catalog No. 11875119, Gibco). Cultures were maintained under standard conditions (37°C and 5% CO_2_), and cells were harvested at D3 and D5 to monitor the dynamics of B cell activation. At each time point, B cell activation markers were quantified by flow cytometry (Table S6), and culture supernatants were also collected to measure the levels of antibodies produced by B cells using enzyme-linked immunosorbent assay (ELISA; Catalog No. 432307, BioLegend, San Diego, CA). The effects of PD-1^high^ and PD-1^low^ Tfh cells on B cell activation and antibody production were statistically evaluated using *t*-test.

### Staining of PBMCs for flow cytometry

PBMCs (1 × 10^6^ cells per sample) were treated with a Cell Activation Cocktail (Catalog No. 423303, BioLegend) and incubated overnight. Cells were then stained using Fixable Viability Dye eFluor 506 (Catalog No. 65-0866-18, Thermo Fisher Scientific) following the manufacturer’s instructions. Cells were then washed with calcium- and magnesium-free PBS containing 2% FBS (Catalog No. A5669701, Thermo Fisher Scientific), and incubated with surface antibodies (detailed in Table S1) for 30 min at 4°C. Following surface staining, cells were fixed and permeabilized using the Foxp3/Transcription Factor Staining Buffer Set (Catalog No. 00-5523-00, Thermo Fisher Scientific). After a 30-min incubation at 4°C, cells were washed and stained with an intracellular marker antibody cocktail in the same buffer for another 30 min at 4°C. Finally, cells were washed thoroughly and resuspended in 100 µl of PBS containing 2% FBS. Cells were subsequently analyzed using a Cytek Aurora full-spectrum flow cytometer (Cytek, CA), and the acquired data were processed and interpreted using the FlowJo software (v10.8.1).

### Statistical analysis

Statistical tools, methods, and significance thresholds for each analysis were explicitly described with the results or detailed either in the figure legends or in the Materials and methods section. The plugins used are listed in Table S1. The *t*-test was used for flow cytometry and SARS-CoV-2 epitope screening data analysis. All statistical analyses of scRNA/V(D)J-seq data were performed using the R software (v4.1).

## Ethical statement

The study protocol was approved by the Ethics Committee of Huashan Hospital, China (Approval No. KY2021-749). All participants provided written informed consent.

## Supplementary Material

qzaf019_Supplementary_Data

## Data Availability

All sequencing data generated in this study have been deposited in the Genome Sequence Archive for Human [42] at the NGDC, CNCB (GSA-Human: HRA002539), and are publicly accessible at https://ngdc.cncb.ac.cn/gsa-human.

## Code availability

The code has been submitted to BioCode at the National Genomics Data Center (NGDC), China National Center for Bioinformation (CNCB) (BioCode: BT007595), which is publicly accessible at https://ngdc.cncb.ac.cn/biocode/tool/BT7595.

## CRediT author statement

**Jingxin Guo:** Conceptualization, Writing – original draft. **Zhangfan Fu:** Methodology, Investigation. **Yi Zhang**: Supervision. **Mengyuan Xu:** Data curation, Formal analysis, Visualization, Writing – review & editing. **Jinhang He:** Resources, Software, Validation, Project administration. **Haocheng Zhang:** Funding acquisition, Resources, Writing – review & editing. **Qiran Zhang:** Investigation, Methodology, Validation. **Jieyu Song:** Formal analysis, Data curation, Writing – original draft. **Ke Lin:** Visualization, Software, Supervision. **Mingxiang Fan:** Project administration, Resources. **Zhangyufan He:** Investigation, Software. **Guanmin Yuan:** Validation, Data curation. **Ning Jiang:** Writing – review & editing, Visualization. **Huang Huang:** Resources, Software. **Chao Qiu:** Supervision, Project administration. **Jingwen Ai:** Writing – review & editing, Visualization. **Wenhong Zhang:** Conceptualization, Methodology, Supervision. All authors have read and approved the final manuscript.

## ORCID

0009-0005-5209-7160 (Jingxin Guo)

0009-0001-1960-2007 (Zhangfan Fu)

0000-0003-0403-7129 (Yi Zhang)

0000-0003-3895-0469 (Mengyuan Xu)

0000-0003-4053-5926 (Jinhang He)

0000-0002-7422-8433 (Haocheng Zhang)

0000-0002-0158-3821 (Qiran Zhang)

0000-0003-4874-3199 (Jieyu Song)

0000-0002-7805-6296 (Ke Lin)

0009-0001-8474-5389 (Mingxiang Fan)

0000-0002-0975-5261 (Zhangyufan He)

0009-0003-3627-3166 (Guanmin Yuan)

0000-0003-0664-6286 (Ning Jiang)

0000-0002-8377-4823 (Huang Huang)

0000-0001-9401-5219 (Chao Qiu)

0000-0002-5152-1557 (Jingwen Ai)

0000-0002-9165-3212 (Wenhong Zhang)
