# Supplementary material for: The High Expression of PD-1 Defines A Subpopulation of Tfh Cells Responding to COVID-19 Vaccine in Humans
Source: Genomics Proteomics Bioinformatics. 2025 Mar 13;23(6):qzaf019. doi: 10.1093/gpbjnl/qzaf019 (PMC13102178; doi:10.1093/gpbjnl/qzaf019)
Supplement: qzaf019_Supplementary_Data [file qzaf019_supplementary_data.zip › Supplementary material captions.docx]

**Supplementary material**

**Figure S1 Comparison of T cell cluster proportions and expression of T helper cell markers in different subtypes**

**A.** The comparison of T cell clusters proportion between InaV and PrSV group (Left), H and L group (right). **B.** Expression of T helper cell markers in different subtypes. **C.** Dotplot of T helper cell markers in different subtypes.

**Figure S2 Flow cytometry gating strategy**

**Figure S3 UMAP analysis of CD3+ T cell marker expression across time points: spectral flow cytometry visualization**

UMAP plots of marker expression for CD3^+^ T cells from each sample collected at D0, D3, D14, and D28 were analyzed by spectral flow cytometry. Regions with high marker expression appear in red, and low in green.

**Figure S4 PD-1^high^ (red) and PD-1^low^ (blue) co-cultured with naive B cells for 3 days, IgG concentration in the culture supernatant**

C, control group.

**Figure S5 Flow cytometry results of PD-1^high^ (red) and PD-1^low^ (blue) co-cultured with naive B cells for 3 days, with group C as the control of naive B cells without Tfh co-culture**

**Figure S6 Identification of Tfh subset cells that highly expressed *LAMP1* and *PDCD1* in COVID-19 infection dataset (PMID: 38898278)**

**A.** UMAP plots of all PBMC cells, color-coded by their broad cell-type annotation. **B.** Barplot of the correlation between each cluster of cells and CD4^+^ Tc5 cells. **C.** Re-cluster of T CD4^+^ follicular helper. **D.** Dotplot of CD4^+^ Tc5 markers in each subcluster of T CD4 follicular helper cells. **E.** Boxplot of C2 subcluster of T CD4 follicular helper in abortive, sustained, and transient infections. COVID-19, coronavirus disease 2019.

**Figure S7 Identification of Tfh subset cells** **that highly expressed *LAMP1 a*nd *PDCD1* in COVID-19 infection dataset (PMID: 33171100)**

**A.** Unsupervised clustering and UMAP of CD4^+^ T cells, colored by cluster ID, blood draw time point, and WOS core levels. **B.** UMAP of CD4^+^ T cells colored by expression of phenotypic markers. **C.** Heatmap of mean expression of CD4^+^ T cell-type typical markers. **D.** Barplot of the correlation between each CD4^+^ T cluster of cells and CD4^+^ Tc5 cells. **E.** Re-cluster of Tfh cell cluster (CD4^+^ Tc4). **F.** Dotplots of CD4^+^ Tc5 markers in each subcluster of CD4^+^ Tc4 cells. **G.** Boxplot of C2 subcluster of CD4^+^ Tc4 cells detected at different blood draw time points in each WOS group. WHO, World Health Organization; WOS, WHO Ordinal Scale.

**Figure S8 Identification of Tfh subset cells that highly expressed *LAMP1* and *PDCD1* in long COVID dataset (PMID: 35216672)**

**A** and **B.** Unsupervised clustering and UMAP of CD4^+^ T cells, colored by cluster ID (**A**) and blood draw time point (**B**). **C.** UMAP of CD4^+^ T cells colored by expression of phenotypic markers. **D.** Heatmap of mean expression of CD4^+^ T cell-type typical markers. **E.** Barplot of the correlation between each CD4^+^ T cluster of cells and CD4^+^ Tc5 cells. **F.** Re-cluster of Tfh cell cluster (CD4^+^ Tc4). **G.** Dotplots of CD4^+^ Tc5 markers in each subcluster of CD4^+^ Tc4 cells. **H.** Boxplot of C2 subcluster of CD4^+^ Tc4 cells detected at different blood draw time points.

**Figure S9 Luciferase activity measurement results for SARS-CoV-2 S and epitope screen**

The comparison of the result of TCR binding with antigen with bright red representing the positive control stimulated by PMA and gray representing the control without antigen stimulation.

**Figure S10 Characterizing CD8^+^ VI-TCR cells during immunization**

**A.** Pie chat revealed the proportion of CD8^+^ VI-TCR cell subsets labeled with cell types. **B.** In each time point mentioned above, the stacked plots were conducted to show the proportion of non-virus-induced TCR cells and VI-TCR cells. **C.** Violin plot showed the program activity score of gene expression patterns labeled with inferred cell subsets between the non-virus-induced TCR cells and VI-TCR cells.

**Figure S11 Comparative expression profiling of cytokine signaling and T cell activation in CD4^+^ TCR cells: highlighting the temporal trends from non-virus-induced to virus-induced states**

The comparisons of the expression score of cytokines-mediated signaling, T cell migration, T cell activation, and IFN-γ production molecule module in non-virus-induced TCR cells and VI-TCR cells within labeled cell populations. We highlighted the violin plots with red rectangle, of which the expression score in mentioned above CD4^+^ cells was peaked on D3/D14 and decreased on D90 significantly.

**Figure S12 Heatmap analysis: enhanced virus-specific cTfh–B cell interactions in high antibody titer groups at D3/D14 and D90**

The heatmap showed that cell–cell interaction between virus-specific activated cTfh cells and B cells were stronger in high antibody titer group versus low antibody titer group at D3/D14 and D90. The relative protein pairs were highlighted with red rectangle.

**Figure S13 Trajectory analysis revealed the overtime shifting of activated cTfh cell phenotypes**

**A.** Pseudotime trajectories for activated cTfh cells phenotypes at D0 (red), D3 (green), D14 (blue), and D90 (purple) post booster vaccination. **B.** Pseudotime trajectories for activated cTfh cells phenotypes, color-coded by the types of TCR clones. Red was the virus-induced clones. Blue was no. While the gray was unknown. **C.** Pseudotime trajectories for activated cTfh cells phenotypes, with color and size coded by the numbers of TCR clones (Left), and further the pseudotime trajectories was divided into four ones according to the time points, D0, D3, D14, and D90. **D.** Distribution of representative clones on the trajectory. **E.** The bubble plot showed the upregulated pathways in branch2, which was analyzed by GOBP enrichment results for gene patterns. GOBP, biological process in Gene Ontology.

**Table S1 Overview of cytometry experimental setup**

**Table S2 Peptide libraries used for stimulation of PBMC and antigen screen**

**Table S3 Overview of validated VI-TCR for SARS-CoV-2 antigen**

**Table S4 Temporal dynamics and cellular distribution of VI-TCR clones in CD4T-5 cluster cells**

**Table S5 The sample information of the cohort**

**Table S6 Flow cytometry antibody for the T and B cells**
