# Supplementary material for: The High Expression of PD-1 Defines A Subpopulation of Tfh Cells Responding to COVID-19 Vaccine in Humans
Source: Genomics Proteomics Bioinformatics. 2025 Mar 13;23(6):qzaf019. doi: 10.1093/gpbjnl/qzaf019 (PMC13102178; doi:10.1093/gpbjnl/qzaf019)
Supplement: qzaf019_Supplementary_Data [file qzaf019_supplementary_data.zip › Table S1.docx]

**Table S1 Overview of cytometry experimental setup**

| **Reagents** | **Source** | **Identifier** |
| --- | --- | --- |
| **Antibodies** |  |  |
| CD98 (BUV496) UM7F8 | BD Pharmingen | 750701 |
| CD3 (BUV661) HIT3a | BD Pharmingen | 741596 |
| CCR7 (BUV563) MAb11 | BD Pharmingen | 749679 |
| TNF (BUV395) MAb11 | BD Pharmingen | 563996 |
| CD279 (BUV615) EH12.1 | BD Pharmingen | 612991 |
| CD25 (BUV737) 2A3 | BD Pharmingen | 612806 |
| CD45RA (BUV805) HI100 | BD Pharmingen | 742020 |
| CD127 (BB515) HIL-7R-M21 | BD Pharmingen | 564423 |
| CCR6 (BB700) 11A9 | BD Pharmingen | 566477 |
| CD57 (PE-CF594) NK-1 | BD Pharmingen | 562488 |
| CD4 (PE-Cy5) RPA-T4 | BD Pharmingen | 555348 |
| T-BET (Alexa Fluor 647) 4B10 | BD Pharmingen | 561264 |
| IL-21 (Alexa Fluor 647) 3A3-N2.1 | BD Pharmingen | 560493 |
| FOXP-3 (R718) 259D/C7 | BD Pharmingen | 566935 |
| IL-4 (V450) 8D4-8 | BD Pharmingen | 561595 |
| CD95 (BV480) DX2 | BD Pharmingen | 746675 |
| CD69 (BV510) FN50 | BD Pharmingen | 747521 |
| CD71 (BV605) M-A712 | BD Pharmingen | 743306 |
| IL-17A (BV650) N49-653 | BD Pharmingen | 563746 |
| CD38 (BV711) HIT2 | BD Pharmingen | 563965 |
| KI-67 (BV786) B56 | BD Pharmingen | 563756 |
| CD107a (FITC) H4A3 | BioLegend | 328606 |
| Granzyme B (APC/Fire750) QA16A02 | BioLegend | 372210 |
| IFN-γ (BV570) 4S.B3 | BioLegend | 502534 |
| CD27 (BV750) O323 | BioLegend | 302850 |
| OX40 (BV421) Ber-ACT35 | BioLegend | 350014 |
| CD8 (PerCP) SK1 | BioLegend | 344708 |
| CXCR5 (APC/Fire810) J252D4 | BioLegend | 356956 |
| BCL-6 (PE/Cyanine7) 7D1 | BioLegend | 358512 |
| IL-2 (PE) MQ1-17H12 | BioLegend | 500307 |
| EOMES (Percp/eFluor^TM^710) WD1928 | Thermo Fisher Scientific | 46-4877-42 |
| Fixable Viability Dye eFluor^TM^ 506 | Thermo Fisher Scientific | 65-0866-18 |
| **Chemicals** |  |  |
| Foxp3/TF Staining Buffer Set | Thermo Fisher Scientific | 00-5523-00 |
| Cell Activation Cocktail (with Brefeldin A) | BioLegend | 423304 |
| **Software and algorithms** |  |  |
| FlowJo 10.8.1 | FlowJo | https://[www.flowjo.com/](http://www.flowjo.com/) |
| UMAP plugin 3.1 | FlowJo | https://[www.flowjo.com/](http://www.flowjo.com/) |
| DownSample 3.3.1 | FlowJo | https://[www.flowjo.com/](http://www.flowjo.com/) |
| GraphPad Prism 8.4.2 | GraphPad Software | https://[www.graphpad.com/](http://www.graphpad.com/) |
