# Supplementary material for: The High Expression of PD-1 Defines A Subpopulation of Tfh Cells Responding to COVID-19 Vaccine in Humans
Source: Genomics Proteomics Bioinformatics. 2025 Mar 13;23(6):qzaf019. doi: 10.1093/gpbjnl/qzaf019 (PMC13102178; doi:10.1093/gpbjnl/qzaf019)
Supplement: qzaf019_Supplementary_Data [file qzaf019_supplementary_data.zip › Table S2.docx]

**Table S2 Peptide libraries used for stimulation of PBMC and antigen screen**

| **Protein** | **Sequence (18AA)** | | | | |
| --- | --- | --- | --- | --- | --- |
| Spike proptein | MFVFLVLLPLVSSQCVNL | NLTTRTQLPPAYTNSFTR | TRGVYYPDKVFRSSVLHS | HSTQDLFLPFFSNVTWFH | FHAIHVSGTNGTKRFDNP |
|  | TNGTKRFDNPVLPFNDGV | GVYFASTEKSNIIRGWIF | IFGTTLDSKTQSLLIVNN | NNATNVVIKVCEFQFCND | NDPFLGVYYHKNNKSWME |
|  | MESEFRVYSSANNCTFEY | EYVSQPFLMDLEGKQGNF | NFKNLREFVFKNIDGYFK | FKIYSKHTPINLVRDLPQ | PQGFSALEPLVDLPIGIN |
|  | INITRFQTLLALHRSYLT | LTPGDSSSGWTAGAAAYY | YYVGYLQPRTFLLKYNEN | ENGTITDAVDCALDPLSE | SETKCTLKSFTVEKGIYQ |
|  | YQTSNFRVQPTESIVRFP | FPNITNLCPFGEVFNATR | TRFASVYAWNRKRISNCV | CVADYSVLYNSASFSTFK | FKCYGVSPTKLNDLCFTN |
|  | TNVYADSFVIRGDEVRQI | QIAPGQTGKIADYNYKLP | LPDDFTGCVIAWNSNNLD | LDSKVGGNYNYLYRLFRK | RKSNLKPFERDISTEIYQ |
|  | YQAGSTPCNGVEGFNCYF | YFPLQSYGFQPTNGVGYQ | YQPYRVVVLSFELLHAPA | PATVCGPKKSTNLVKNKC | KCVNFNFNGLTGTGVLTE |
|  | TESNKKFLPFQQFGRDIA | IADTTDAVRDPQTLEILD | LDITPCSFGGVSVITPGT | GTNTSNQVAVLYQDVNCT | CTEVPVAIHADQLTPTWR |
|  | WRVYSTGSNVFQTRAGCL | CLIGAEHVNNSYECDIPI | PIGAGICASYQTQTNSPR | PRRARSVASQSIIAYTMS | MSLGAENSVAYSNNSIAI |
|  | AIPTNFTISVTTEILPVS | VSMTKTSVDCTMYICGDS | DSTECSNLLLQYGSFCTQ | TQLNRALTGIAVEQDKNT | NTQEVFAQVKQIYKTPPI |
|  | PIKDFGGFNFSQILPDPS | PSKPSKRSFIEDLLFNKV | KVTLADAGFIKQYGDCLG | LGDIAARDLICAQKFNGL | GLTVLPPLLTDEMIAQYT |
|  | YTSALLAGTITSGWTFGA | GAGAALQIPFAMQMAYRF | RFNGIGVTQNVLYENQKL | KLIANQFNSAIGKIQDSL | SLSSTASALGKLQDVVNQ |
|  | NQNAQALNTLVKQLSSNF | NFGAISSVLNDILSRLDK | DKVEAEVQIDRLITGRLQ | LQSLQTYVTQQLIRAAEI | EIRASANLAATKMSECVL |
|  | VLGQSKRVDFCGKGYHLM | LMSFPQSAPHGVVFLHVT | VTYVPAQEKNFTTAPAIC | ICHDGKAHFPREGVFVSN | SNGTHWFVTQRNFYEPQI |
|  | QIITTDNTFVSGNCDVVI | VIGIVNNTVYDPLQPELD | LDSFKEELDKYFKNHTSP | SPDVDLGDISGINASVVN | VNIQKEIDRLNEVAKNLN |
|  | LNESLIDLQELGKYEQYI | YIKWPWYIWLGFIAGLIA | IAIVMVTIMLCCMTSCCS | CSCLKGCCSCGSCCKFDE | DEDDSEPVLKGVKLHYT |
|  | PLVSSQCVNLTTRTQLPP | PPAYTNSFTRGVYYPDKV | KVFRSSVLHSTQDLFLPF | PFFSNVTWFHAIHVSGTN | YHKNNKSWMESEFRVYSS |
|  | NPVLPFNDGVYFASTEKS | KSNIIRGWIFGTTLDSKT | KTQSLLIVNNATNVVIKV | KVCEFQFCNDPFLGVYYH | PLVDLPIGINITRFQTLL |
|  | SSANNCTFEYVSQPFLMD | MDLEGKQGNFKNLREFVF | VFKNIDGYFKIYSKHTPI | PINLVRDLPQGFSALEPL | SFTVEKGIYQTSNFRVQP |
|  | LLALHRSYLTPGDSSSGW | GWTAGAAAYYVGYLQPRT | RTFLLKYNENGTITDAVD | VDCALDPLSETKCTLKSF | TKLNDLCFTNVYADSFVI |
|  | QPTESIVRFPNITNLCPF | PFGEVFNATRFASVYAWN | WNRKRISNCVADYSVLYN | YNSASFSTFKCYGVSPTK | ERDISTEIYQAGSTPCNG |
|  | VIRGDEVRQIAPGQTGKI | KIADYNYKLPDDFTGCVI | VIAWNSNNLDSKVGGNYN | YNYLYRLFRKSNLKPFER | GLTGTGVLTESNKKFLPF |
|  | NGVEGFNCYFPLQSYGFQ | FQPTNGVGYQPYRVVVLS | LSFELLHAPATVCGPKKS | KSTNLVKNKCVNFNFNGL | HADQLTPTWRVYSTGSNV |
|  | PFQQFGRDIADTTDAVRD | RDPQTLEILDITPCSFGG | GGVSVITPGTNTSNQVAV | AVLYQDVNCTEVPVAIHA | VAYSNNSIAIPTNFTISV |
|  | NVFQTRAGCLIGAEHVNN | NNSYECDIPIGAGICASY | SYQTQTNSPRRARSVASQ | SQSIIAYTMSLGAENSVA | VKQIYKTPPIKDFGGFNF |
|  | SVTTEILPVSMTKTSVDC | DCTMYICGDSTECSNLLL | LLQYGSFCTQLNRALTGI | GIAVEQDKNTQEVFAQVK | LTDEMIAQYTSALLAGTI |
|  | NFSQILPDPSKPSKRSFI | FIEDLLFNKVTLADAGFI | FIKQYGDCLGDIAARDLI | LICAQKFNGLTVLPPLLT | LGKLQDVVNQNAQALNTL |
|  | TITSGWTFGAGAALQIPF | PFAMQMAYRFNGIGVTQN | QNVLYENQKLIANQFNSA | SAIGKIQDSLSSTASALG | AATKMSECVLGQSKRVDF |
|  | TLVKQLSSNFGAISSVLN | LNDILSRLDKVEAEVQID | IDRLITGRLQSLQTYVTQ | TQQLIRAAEIRASANLAA | TQRNFYEPQIITTDNTFV |
|  | DFCGKGYHLMSFPQSAPH | PHGVVFLHVTYVPAQEKN | KNFTTAPAICHDGKAHFP | FPREGVFVSNGTHWFVTQ | RLNEVAKNLNESLIDLQE(17AA) |
|  | FVSGNCDVVIGIVNNTVY | VYDPLQPELDSFKEELDK | DKYFKNHTSPDVDLGDIS | ISGINASVVNIQKEIDRL | / |
|  | QELGKYEQYIKWPWYIWL | WLGFIAGLIAIVMVTIML | MLCCMTSCCSCLKGCCSC | SCGSCCKFDEDDSEPVLK | / |
| N Protein | MSDNGPQNQRNAPRITFG | RATRRIRGGDGKMKDLSP | RGGSQASSRSSSRSRNSS | TKAYNVTQAFGRRGPEQT | LNKHIDAYKTFPPTEPKK |
|  | QRNAPRITFGGPSDSTGS | GDGKMKDLSPRWYFYYLG | RSSSRSRNSSRNSTPGSS | AFGRRGPEQTQGNFGDQE | KTFPPTEPKKDKKKKADE |
|  | FGGPSDSTGSNQNGERSG | SPRWYFYYLGTGPEAGLP | SSRNSTPGSSRGTSPARM | QTQGNFGDQELIRQGTDY | KKDKKKKADETQALPQRQ |
|  | GSNQNGERSGARSKQRRP | LGTGPEAGLPYGANKDGI | SSRGTSPARMAGNGGDAA | QELIRQGTDYKHWPQIAQ | DETQALPQRQKKQQTVTL |
|  | SGARSKQRRPQGLPNNTA | LPYGANKDGIIWVATEGA | RMAGNGGDAALALLLLDR | DYKHWPQIAQFAPSASAF | RQKKQQTVTLLPAADLDD |
|  | RPQGLPNNTASWFTALTQ | GIIWVATEGALNTPKDHI | AALALLLLDRLNQLESKM | AQFAPSASAFFGMSRIGM | TLLPAADLDDFSKQLQQS |
|  | TASWFTALTQHGKEDLKF | GALNTPKDHIGTRNPANN | DRLNQLESKMSGKGQQQQ | AFFGMSRIGMEVTPSGTW | DDFSKQLQQSMSSADSTQ |
|  | TQHGKEDLKFPRGQGVPI | HIGTRNPANNAAIVLQLP | KMSGKGQQQQGQTVTKKS | GMEVTPSGTWLTYTGAIK | / |
|  | KFPRGQGVPINTNSSPDD | NNAAIVLQLPQGTTLPKG | QQGQTVTKKSAAEASKKP | TWLTYTGAIKLDDKDPNF | / |
|  | PINTNSSPDDQIGYYRRA | LPQGTTLPKGFYAEGSRG | KSAAEASKKPRQKRTATK | IKLDDKDPNFKDQVILLN | / |
|  | DDQIGYYRRATRRIRGGD | KGFYAEGSRGGSQASSRS | KPRQKRTATKAYNVTQAF | NFKDQVILLNKHIDAYKT | / |

*Note*: PBMC, peripheral blood mononuclear cell; AA, amino acid.
