# Supplementary material for: The High Expression of PD-1 Defines A Subpopulation of Tfh Cells Responding to COVID-19 Vaccine in Humans
Source: Genomics Proteomics Bioinformatics. 2025 Mar 13;23(6):qzaf019. doi: 10.1093/gpbjnl/qzaf019 (PMC13102178; doi:10.1093/gpbjnl/qzaf019)
Supplement: qzaf019_Supplementary_Data [file qzaf019_supplementary_data.zip › Table S3.docx]

| **Index** | **Sample** | **Type** | **ulTcRb** | **TcRb** | **V** | **J** | **TcRa** | **HLA-A** | **Frequency** |
| --- | --- | --- | --- | --- | --- | --- | --- | --- | --- |
| Group 1 | 480 | global-S%WTGNTE | CASSQwTGNTEAFF | CASSQWTGNTEAFF | TRBV11-2 | TRBJ1-1 | CAGASNNAGNMLTF | A*02:03:01/A*11:01:01 | 110 |
| Group 1 | database1788 | global-S%WTGNTE | CASSEwTGNTEAFF | CASSEWTGNTEAFF | TRBV5-1 | TRBJ1-1 | CAVRGVTGGGNKLTF | A*02:01 | / |
| Group 2 | database3211 | global-SL%GATNEK | CASSLdgaTNEKLFF | CASSLDGATNEKLFF | TRBV5-1 | TRBJ1-4 | CAVGARSGGYQKVTF | A*02:01 | / |
| Group 2 | 518 | global-SL%GATNEK | CASSLwgaTNEKLFF | CASSLWGATNEKLFF | TRBV28 | TRBJ1-4 | CALLYNAGNNRKLIW | A*02:07:01/A*30:01:01 | 5 |
| Group 3 | database1524 | global-SP%TDT | CASSPgTDTQYF | CASSPGTDTQYF | TRBV5-1 | TRBJ2-3 | CAVGVDTGTASKLTF | A*02:01 | / |
| Group 3 | 620 | global-SP%TDT | CASSPrTDTQYF | CASSPRTDTQYF | TRBV7-9 | TRBJ2-3 | CAVREAGGYQKVTF | A*02:03:01/A*11:01:01 | 11 |
| Group 4 | database3894 | motif-EENT | CASSEeNTGELFF | CASSEENTGELFF | TRBV9 | TRBJ2-2 | CAEPSSASKIIF | A*02:01 | / |
| Group 4 | database901 | motif-EENT | CASneeNTGELFF | CASNEENTGELFF | TRBV5-1 | TRBJ2-2 | CVVNNNNDMRF | A*02:01 | / |
| Group 4 | database3158 | motif-EENT | CASneeNTGELFF | CASNEENTGELFF | TRBV5-1 | TRBJ2-2 | CVVNNNNDMRF | A*02:01 | / |
| Group 4 | database3679 | motif-EENT | CASGeeNTGELFF | CASGEENTGELFF | TRBV5-1 | TRBJ2-2 | / | A*02 | / |
| Group 4 | database3519 | motif-EENT | CATSeeNTGELFF | CATSEENTGELFF | TRBV24-1 | TRBJ2-2 | / | A*02 | / |
| Group 4 | database1443 | motif-EENT | CASmeeNTGELFF | CASMEENTGELFF | TRBV5-1 | TRBJ2-2 | CVVNRDNDMRF | A*02:01 | / |
| Group 4 | database3645 | motif-EENT | CASmeeNTGELFF | CASMEENTGELFF | TRBV5-1 | TRBJ2-2 | CVVNRDNDMRF | A*02 | / |
| Group 4 | 154 | motif-EENT | CATSDLeeNTGELFF | CATSDLEENTGELFF | TRBV24-1 | TRBJ2-2 | CAVNARFDDYKLSF | A*02:07:01/A*30:01:01 | 1 |

**Table S3 Overview of validated VI-TCR for SARS-CoV-2 antigen**

*Note*: VI-TCR, virus-induced TCR; V, variable; J, joining; TCR, T cell receptor. TcRa，T-cell receptor alpha；TcRb, T-cell Receptor beta; ulTcRb, amino acids encoded by codon overlapping with N-addition nucleotides are labeled as lower case, otherwise, it is labeled as upper cases.
