# Supplementary material for: The High Expression of PD-1 Defines A Subpopulation of Tfh Cells Responding to COVID-19 Vaccine in Humans
Source: Genomics Proteomics Bioinformatics. 2025 Mar 13;23(6):qzaf019. doi: 10.1093/gpbjnl/qzaf019 (PMC13102178; doi:10.1093/gpbjnl/qzaf019)
Supplement: qzaf019_Supplementary_Data [file qzaf019_supplementary_data.zip › Table S4.docx]

| **Clone** | **Time point** | **Cell type** |
| --- | --- | --- |
| TRAV10: CVVSVSGSAROLTF.TRAJ22.  TRBV7-2:C ASSSGGGGGYTF.TRBJ1-2 | D3/D14 | CD4 T-5 |
| TRAV8.2: CAUKNPCGSOGNLIF.TRAJ42.  TRBV7.9: CASSLTGDYGYTF.TRBJ1-2 | D3/D14 | CD4 T-5 |

**Table S4 Temporal dynamics and cellular distribution of VI-TCR clones in CD4 T-5 cluster cells**
