# Supplementary material for: The High Expression of PD-1 Defines A Subpopulation of Tfh Cells Responding to COVID-19 Vaccine in Humans
Source: Genomics Proteomics Bioinformatics. 2025 Mar 13;23(6):qzaf019. doi: 10.1093/gpbjnl/qzaf019 (PMC13102178; doi:10.1093/gpbjnl/qzaf019)
Supplement: qzaf019_Supplementary_Data [file qzaf019_supplementary_data.zip › Table S6.docx]

| **Table S6 Flow cytometry antibody for the T and B cells** |  |  |
| --- | --- | --- |
| **Antibody** | **Souce** | **Catalog number** |
| Alexa Fluor 700 (CD19) HIB19 | BD Pharmingen | 557921 |
| BV510 (CD38) HIT2 | BD Pharmingen | 563251 |
| BV650 (PD-1) EH12.1 | BD Pharmingen | 564104 |
| Alexa Fluor 647 (CCL5) 2D5 | BD Pharmingen | 515506 |
| BV605 (IgM) G20-127 | BD Pharmingen | 562977 |
| BV480 (IgD) IA6-2 | BD Pharmingen | 566138 |
| BV421 (IgG) G18-145 | BD Pharmingen | 562581 |
| FITC (CD4) RPA-T4 | BD Pharmingen | 300506 |
| BV750 (CD27) O323 | BD Pharmingen | 302850 |
| BB700 (CCR6) 11A9 | BD Pharmingen | 566477 |
| FITC (CD107a) H4A3 | BioLegend | 328608 |
| CXCR5 (PE/Cyanine7) J252D4 | BioLegend | 356924 |
| APC-Cy7 Zombie NIR | BioLegend | 423105 |
