# Supplementary figures and images for: The High Expression of PD-1 Defines A Subpopulation of Tfh Cells Responding to COVID-19 Vaccine in Humans
Source: Genomics Proteomics Bioinformatics. 2025 Mar 13;23(6):qzaf019. doi: 10.1093/gpbjnl/qzaf019 (PMC13102178; doi:10.1093/gpbjnl/qzaf019)

A

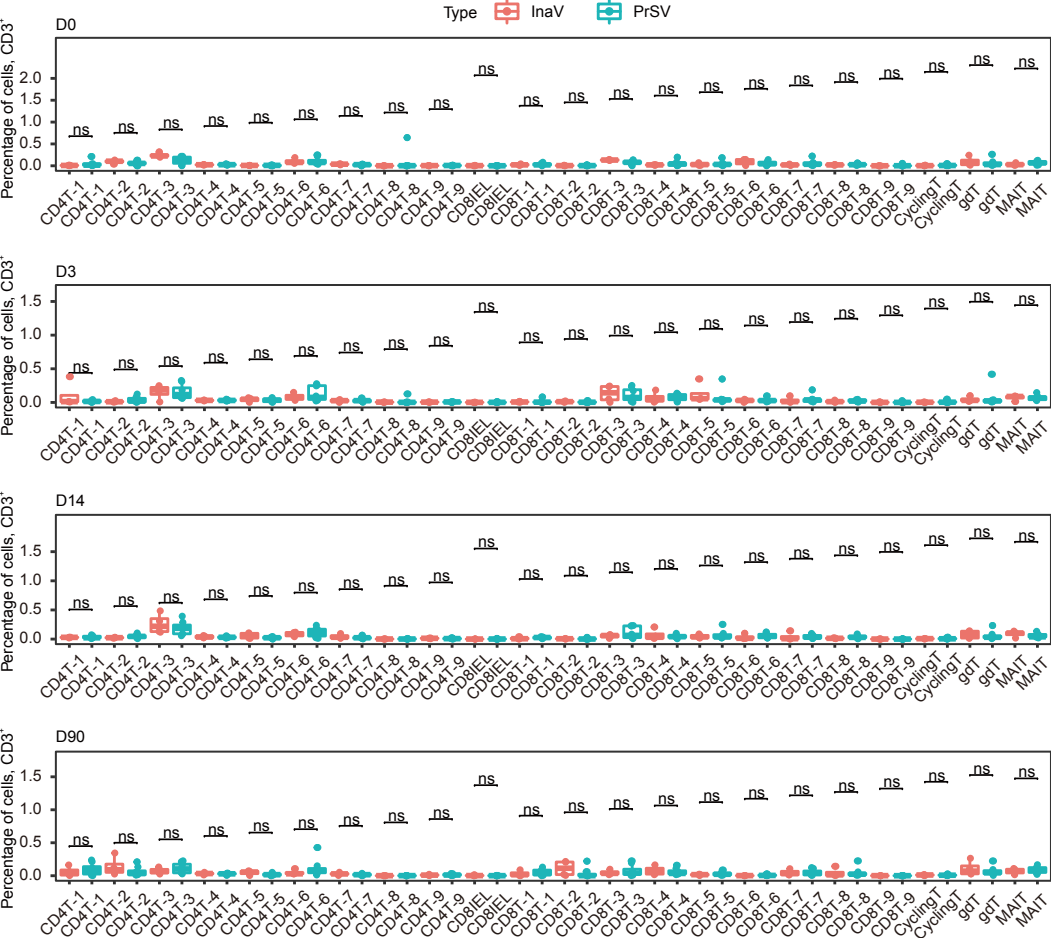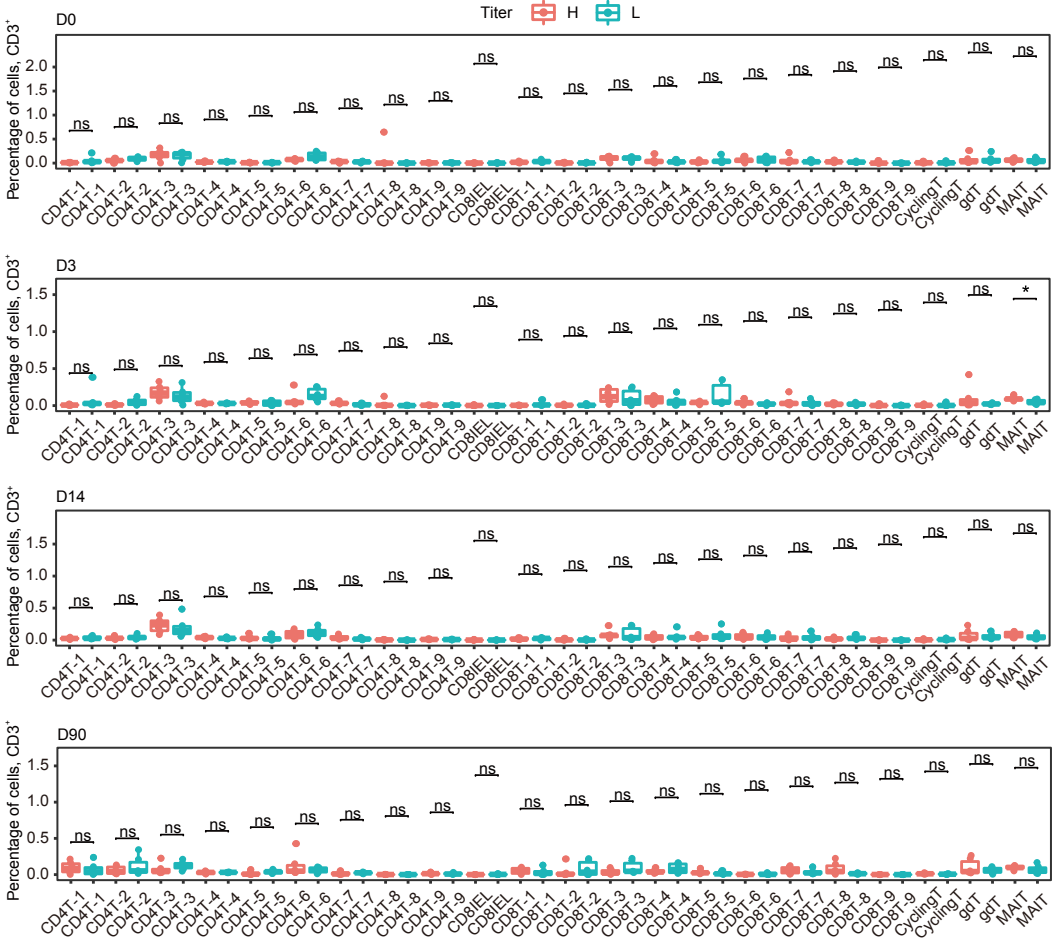

B

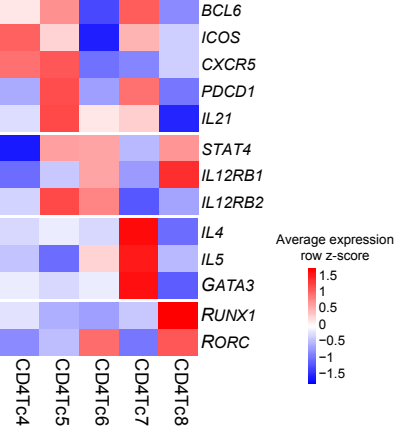

C

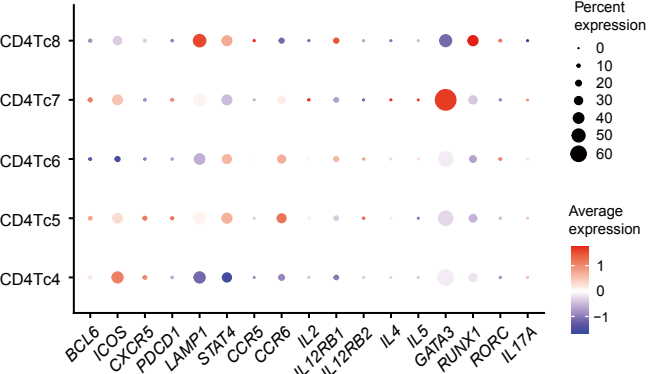

Supplement: qzaf019_Supplementary_Data [file qzaf019_supplementary_data.zip › Figure S1.pdf]

A

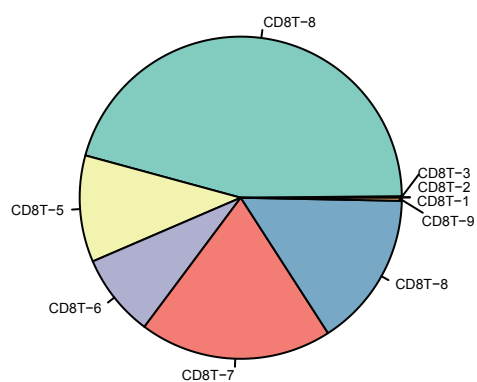

B

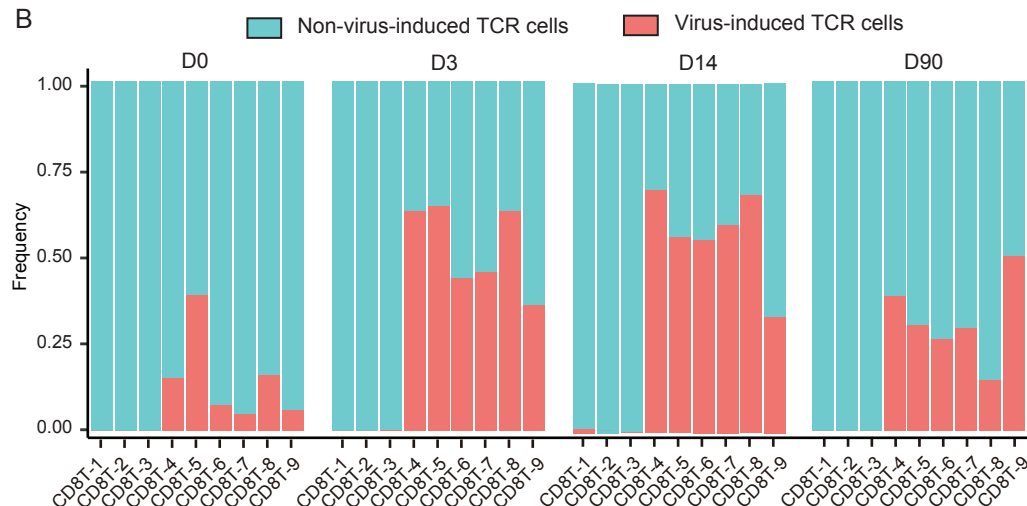

C

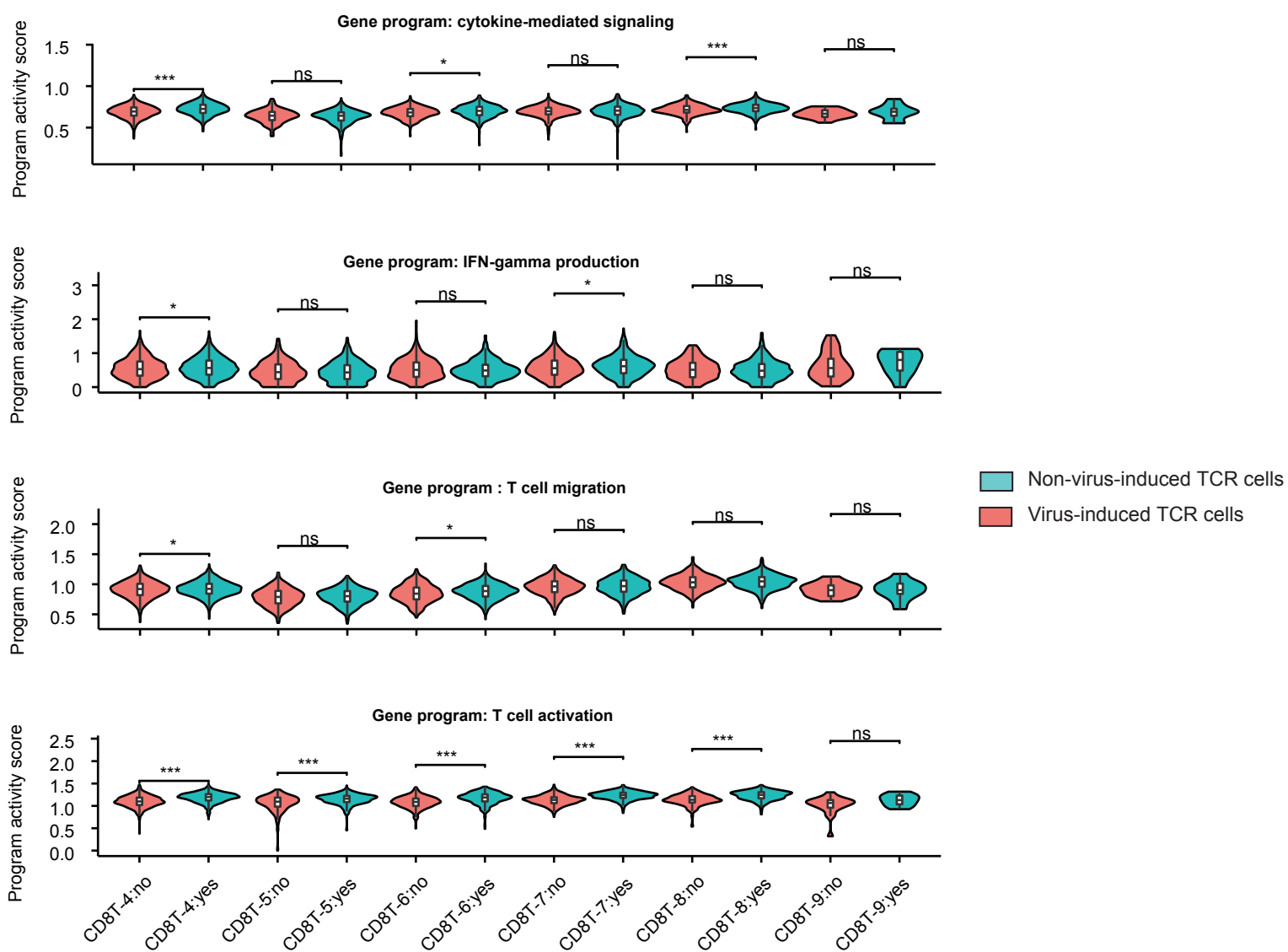

Supplement: qzaf019_Supplementary_Data [file qzaf019_supplementary_data.zip › Figure S10.pdf]

Non-virus-induced TCR cells Virus-induced TCR cells

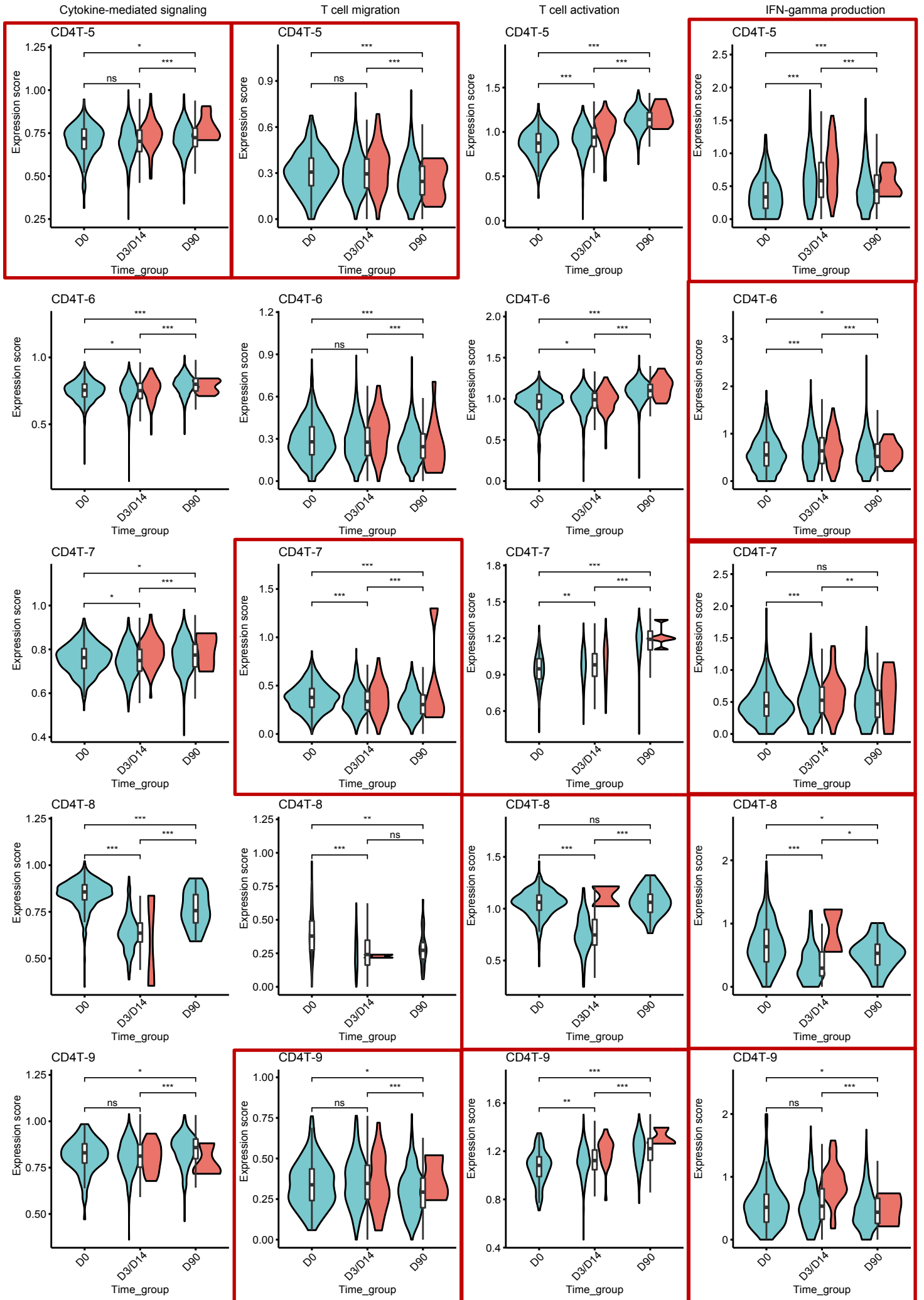

Supplement: qzaf019_Supplementary_Data [file qzaf019_supplementary_data.zip › Figure S11.pdf]

Supplementary Figure 12

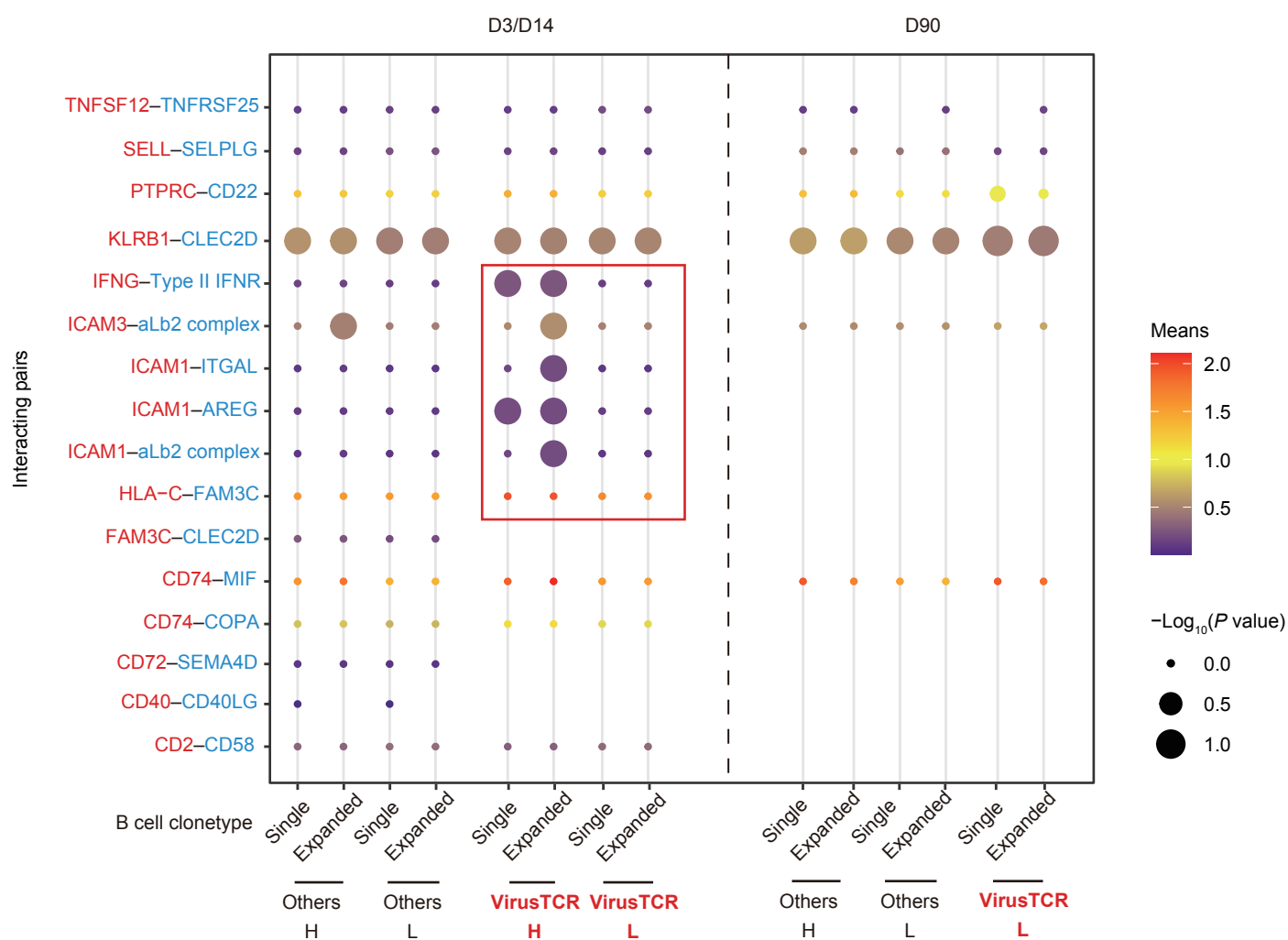

Supplement: qzaf019_Supplementary_Data [file qzaf019_supplementary_data.zip › Figure S12.pdf]

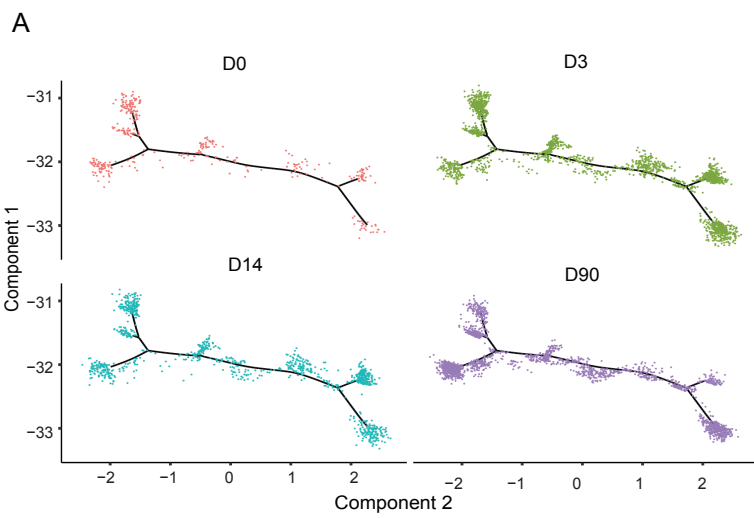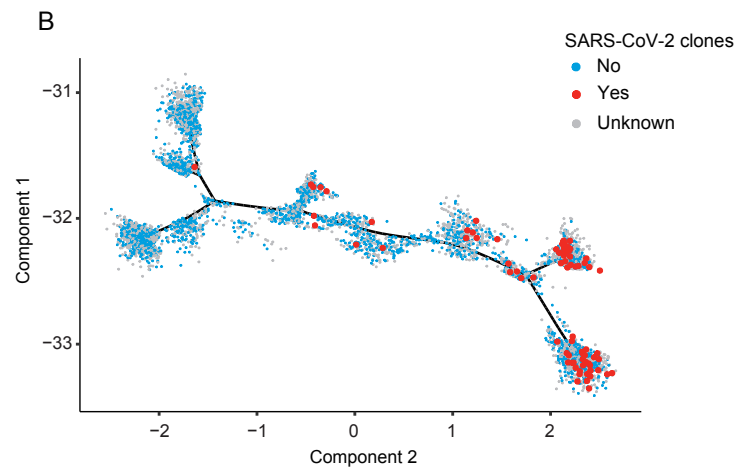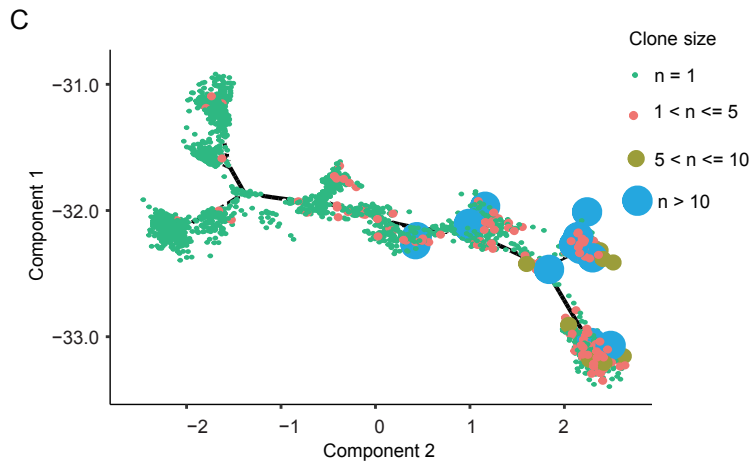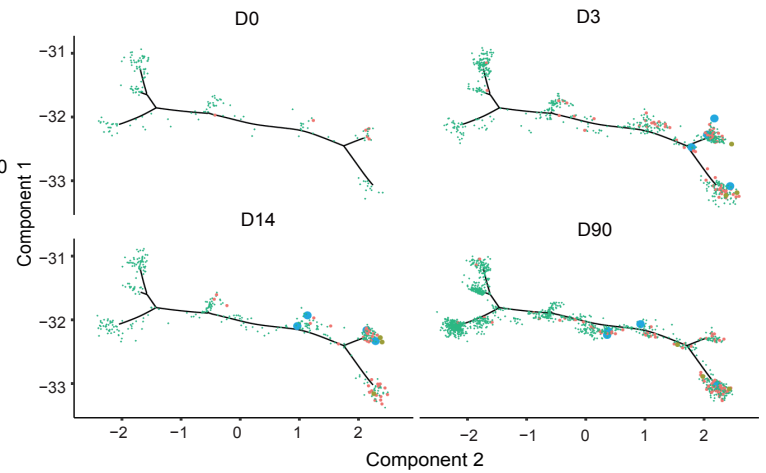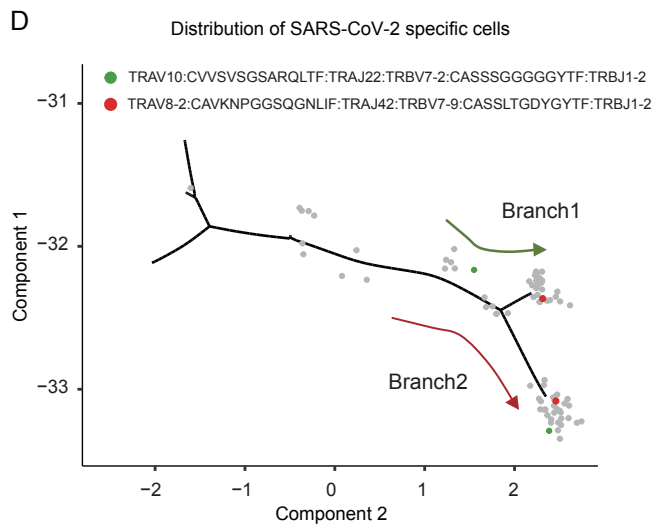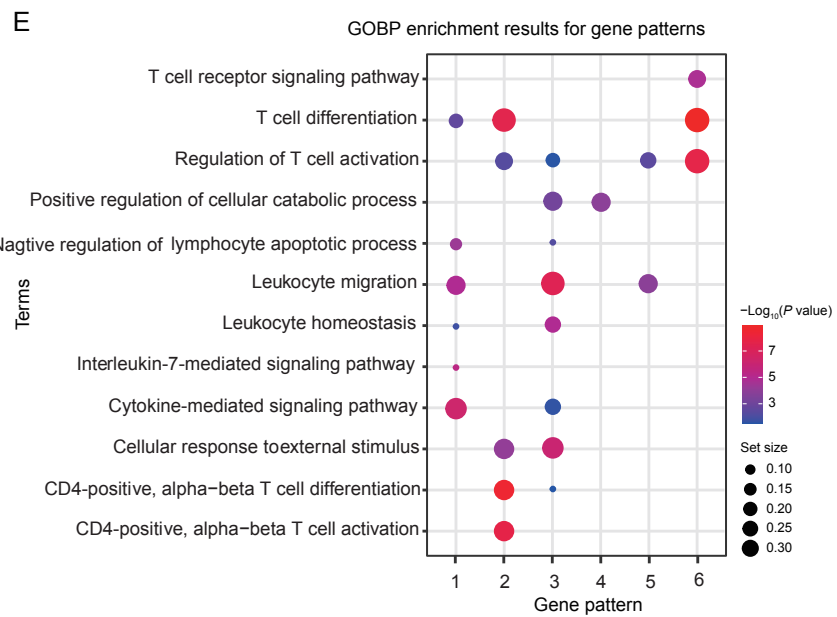

Supplement: qzaf019_Supplementary_Data [file qzaf019_supplementary_data.zip › Figure S13.pdf]

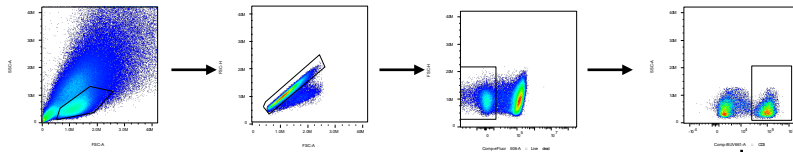

**CD8**

**CD4**

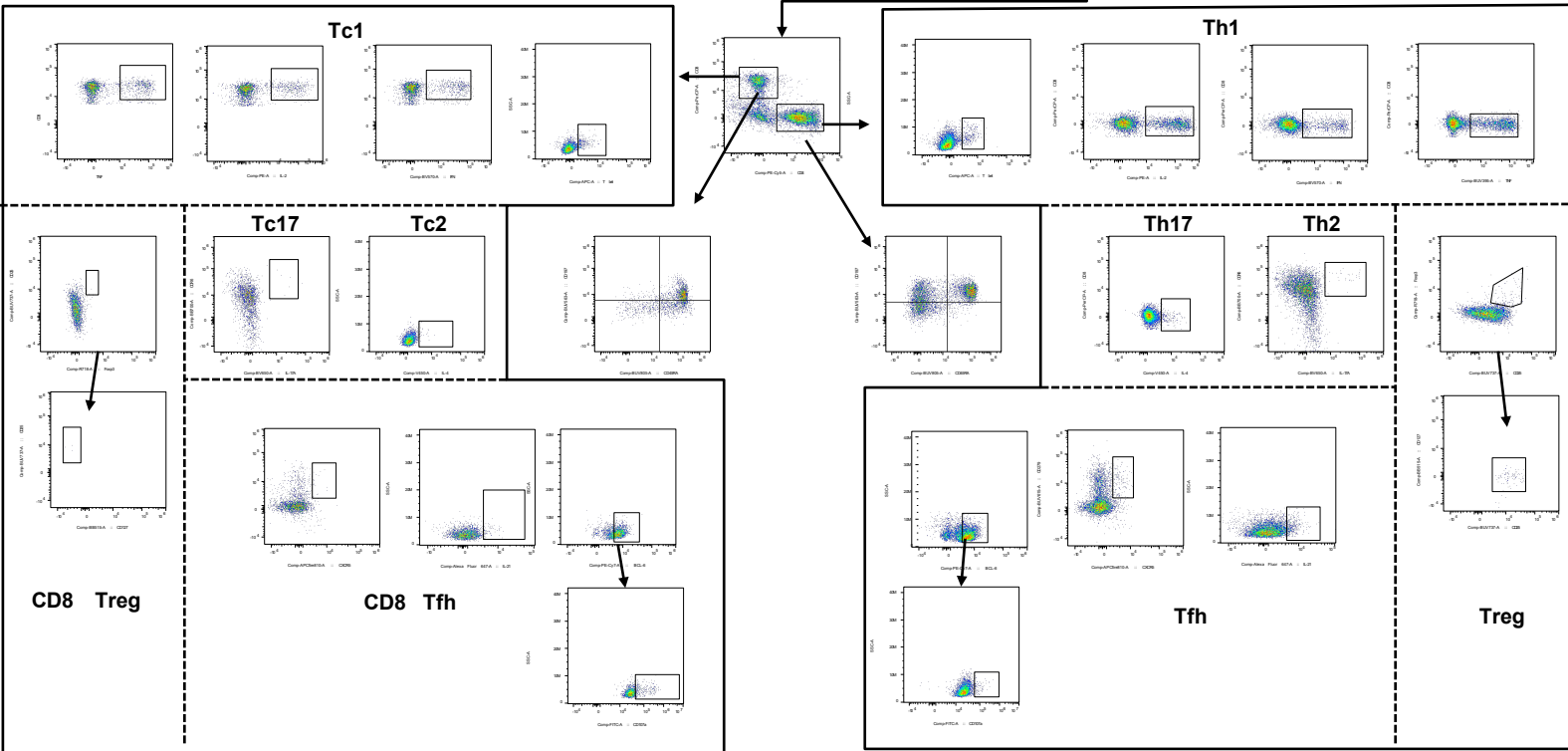

Supplement: qzaf019_Supplementary_Data [file qzaf019_supplementary_data.zip › Figure S2.pdf]

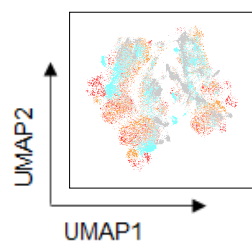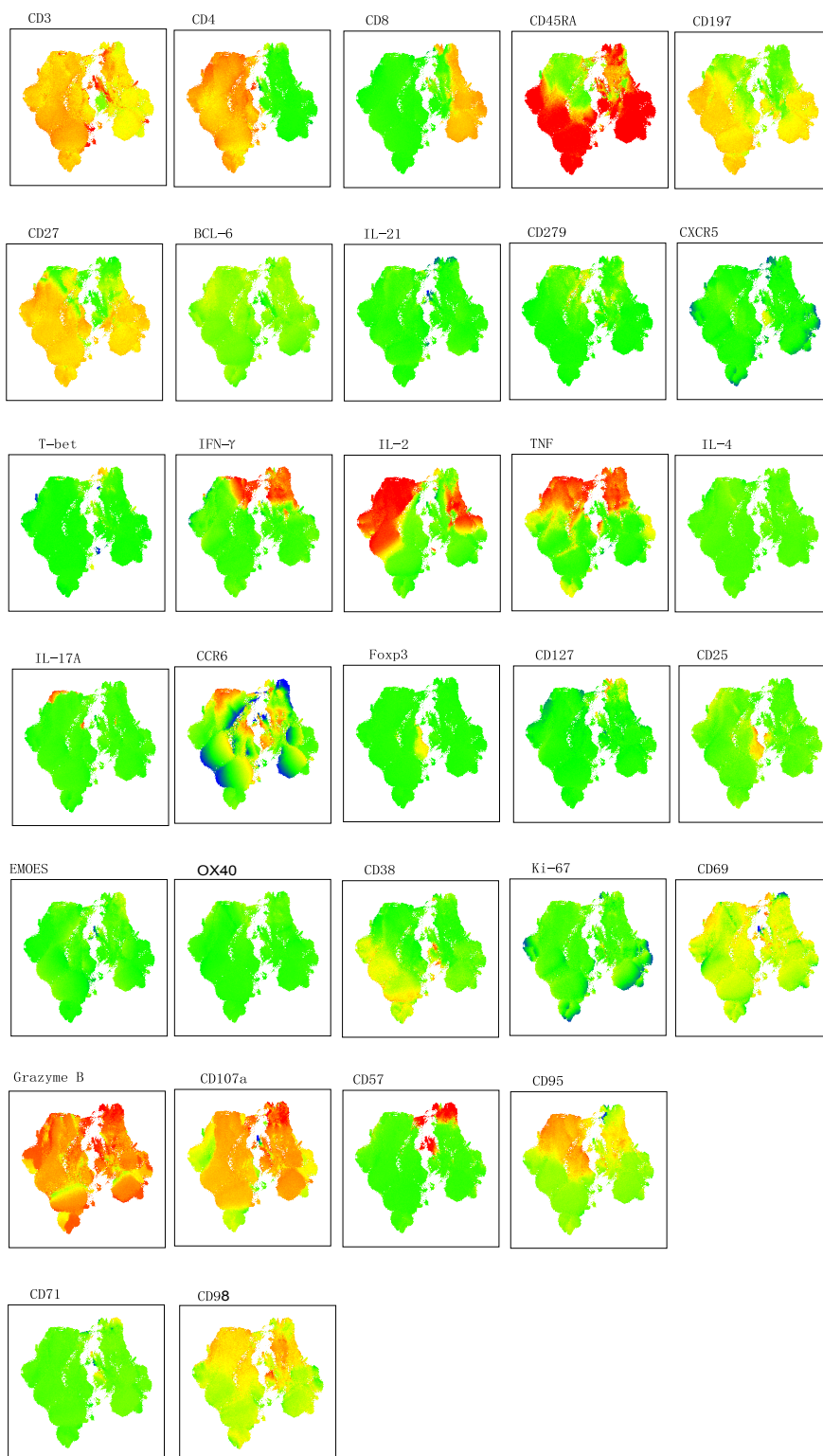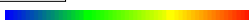

Supplement: qzaf019_Supplementary_Data [file qzaf019_supplementary_data.zip › Figure S3.pdf]

# IgG

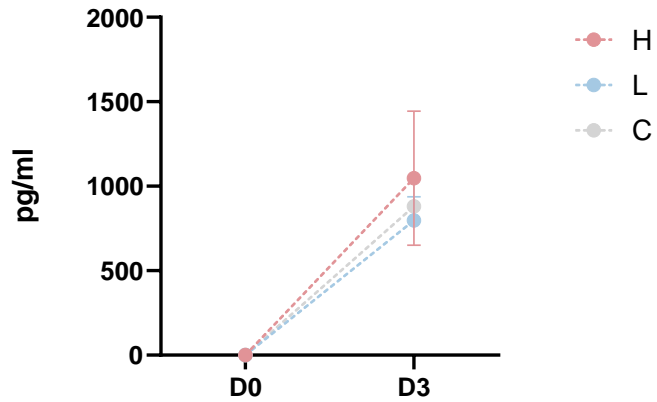

Supplement: qzaf019_Supplementary_Data [file qzaf019_supplementary_data.zip › Figure S4.pdf]

**CD19/CD38**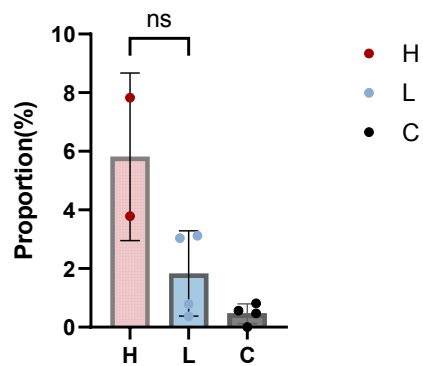**CD19/IgG**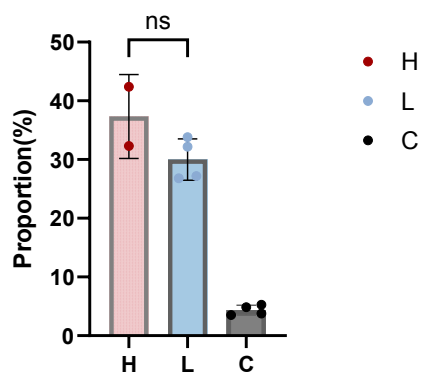**CD19/IgM**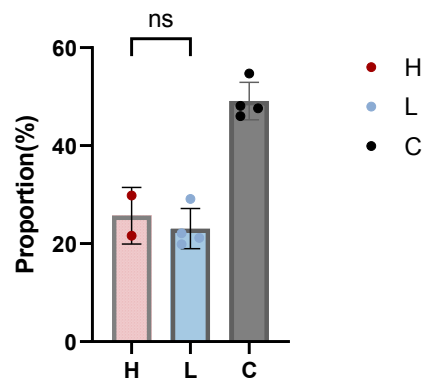**CD19/IgM, IgG**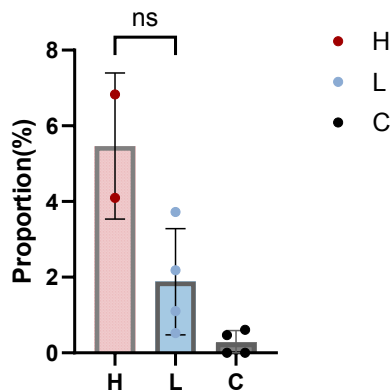**CD19/Memory B**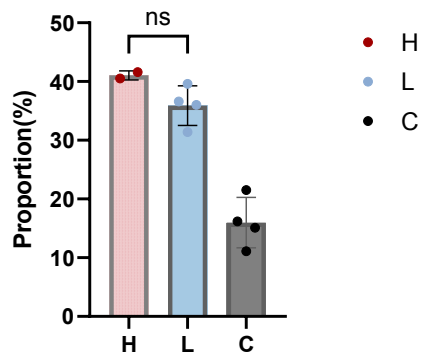**CD19/Plasma B**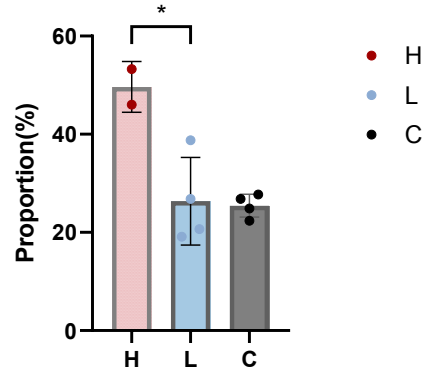**Tfh/CD107a**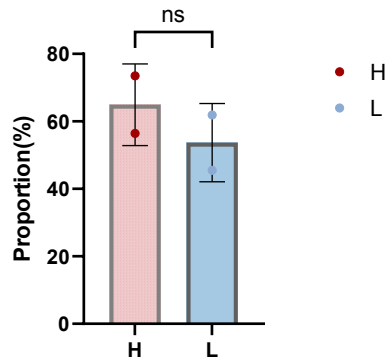**Tfh/CCR6**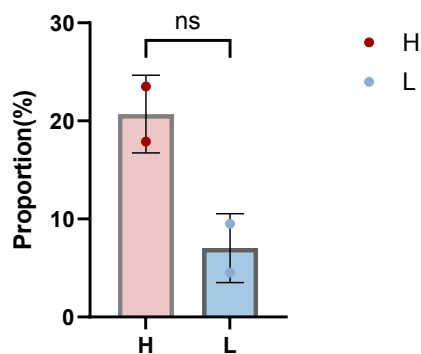**Tfh/CCL5**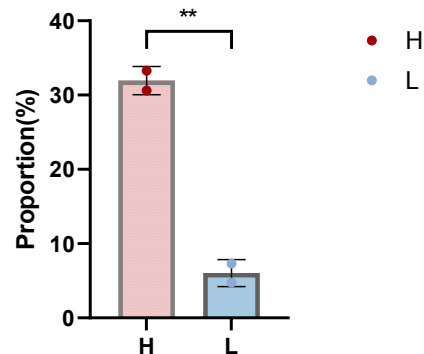

Supplement: qzaf019_Supplementary_Data [file qzaf019_supplementary_data.zip › Figure S5.pdf]

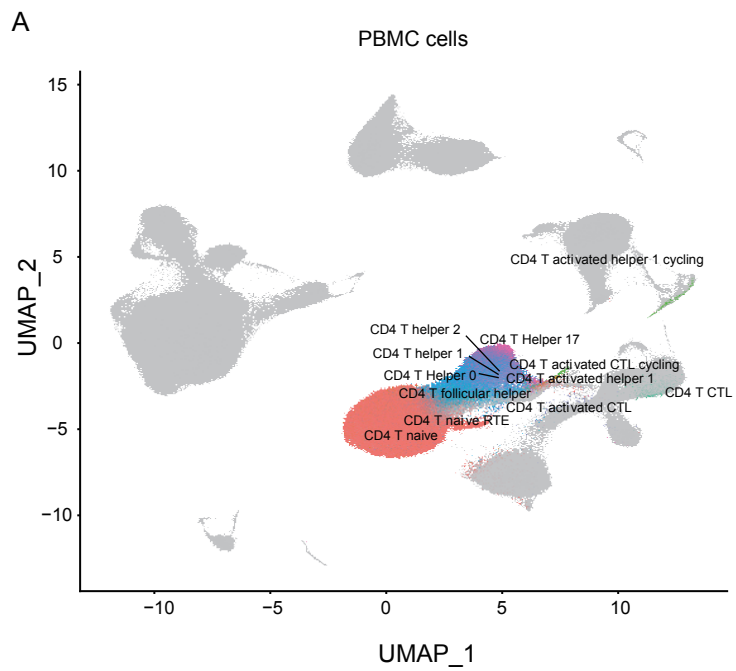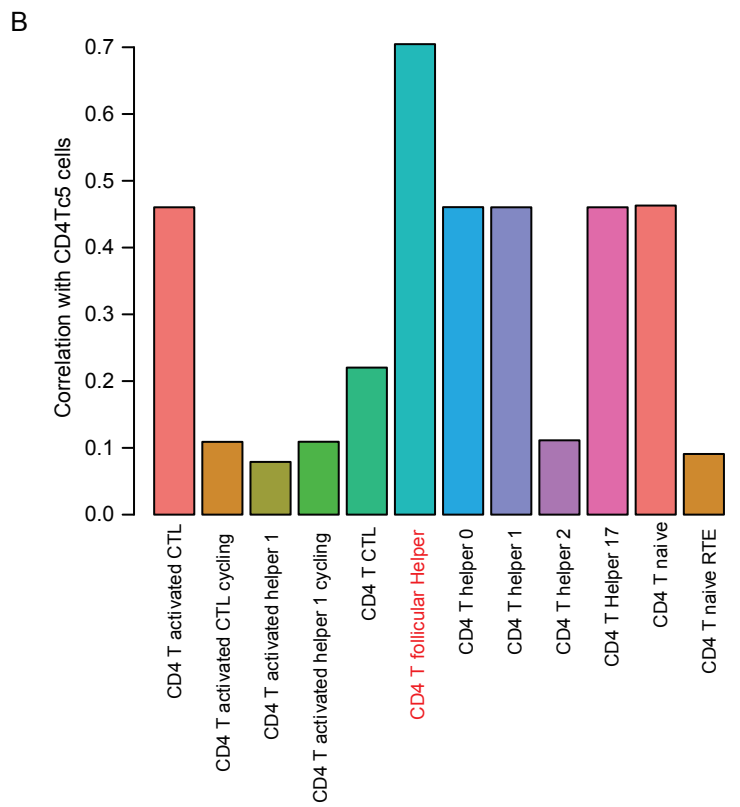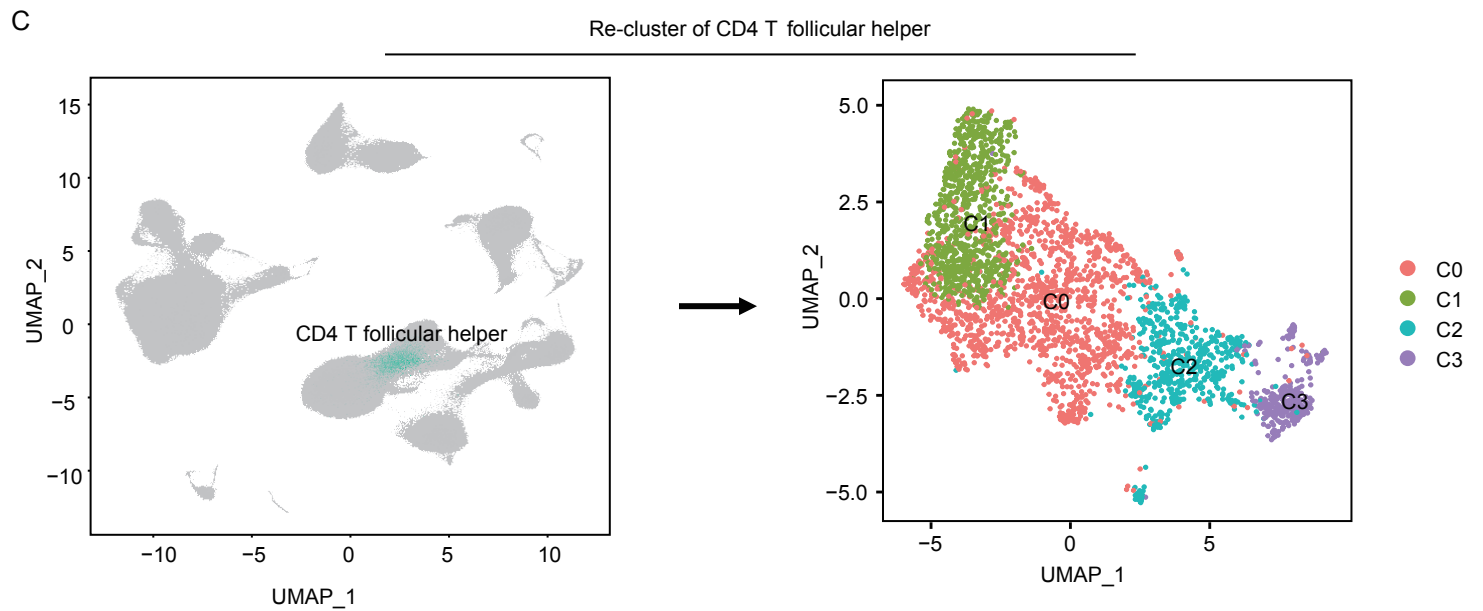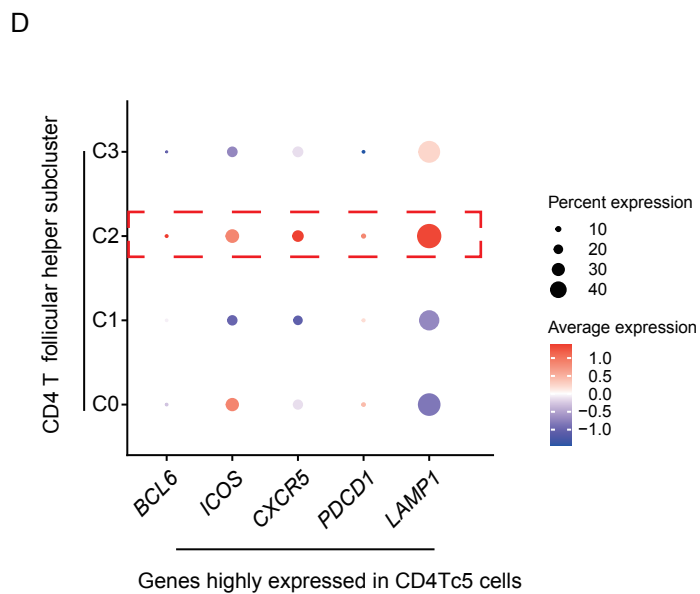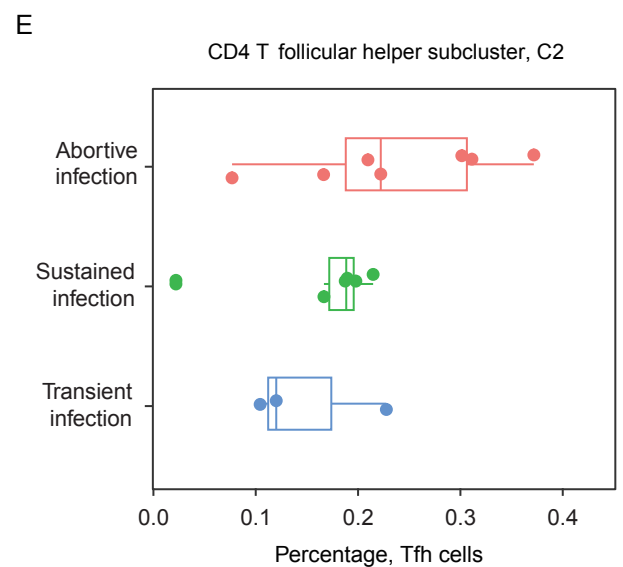

Supplement: qzaf019_Supplementary_Data [file qzaf019_supplementary_data.zip › Figure S6.pdf]

A

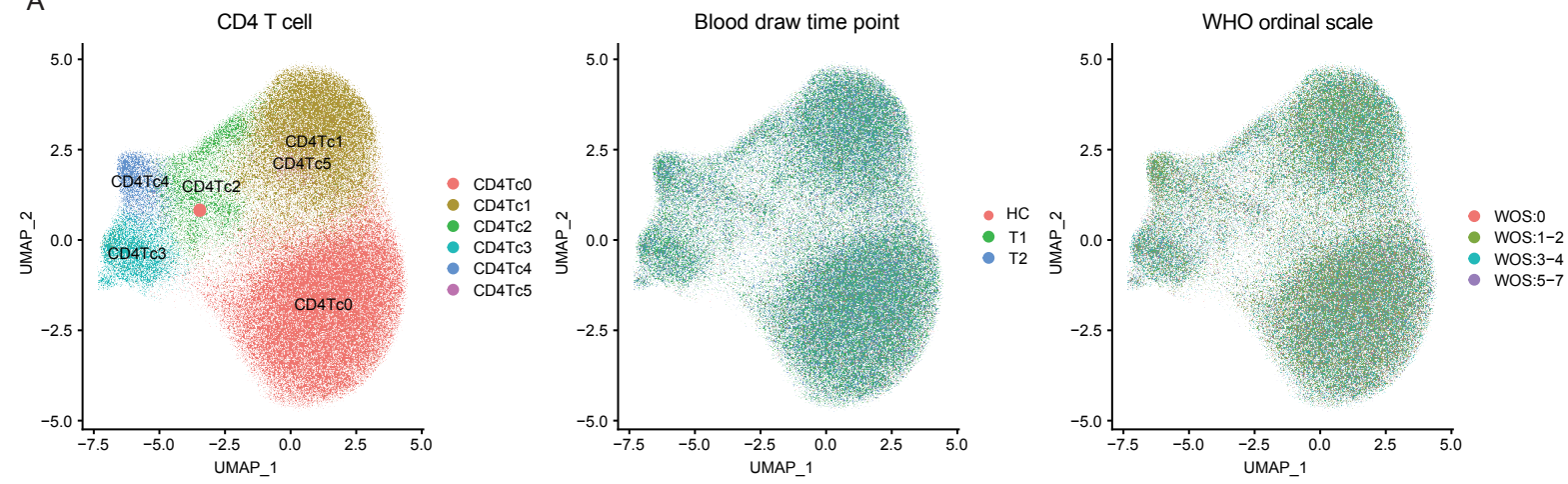

B

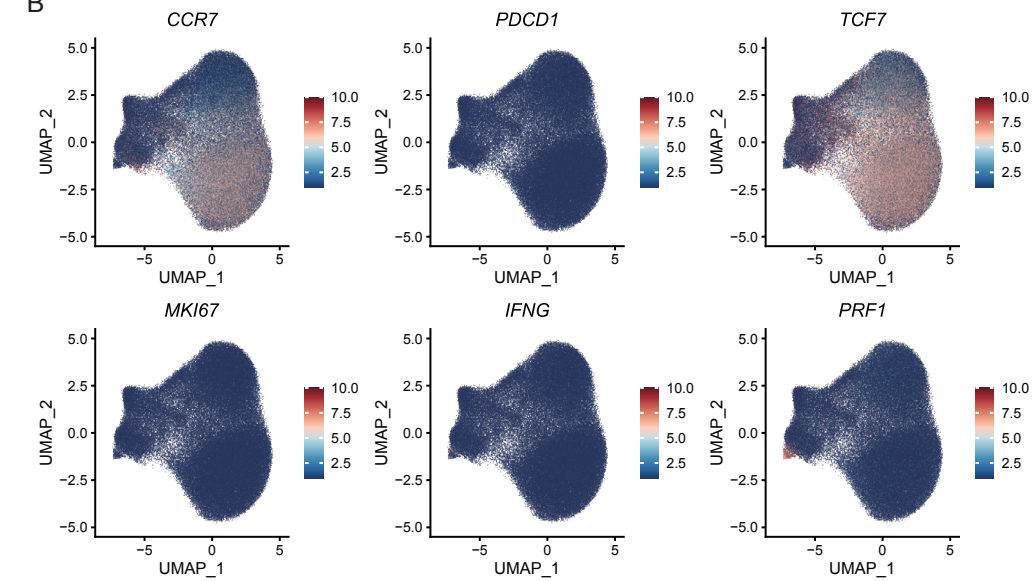

C

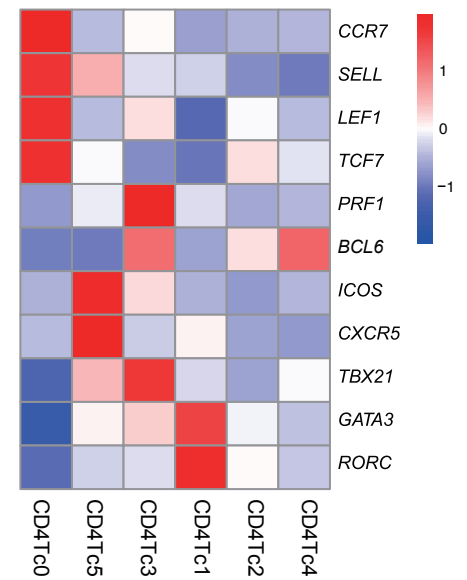

D

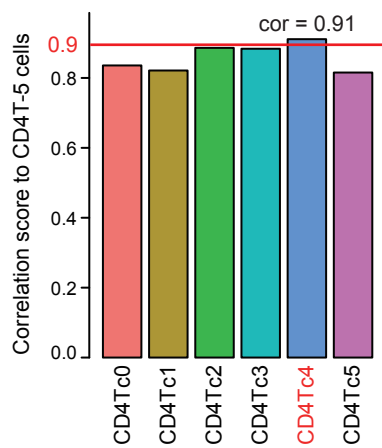

E

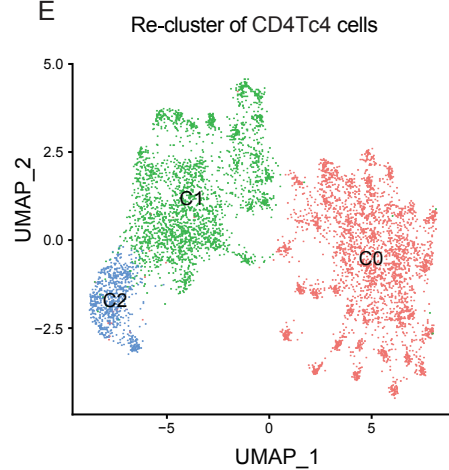

F

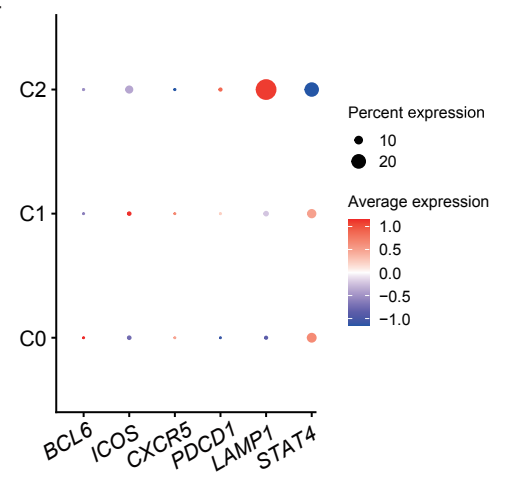

G

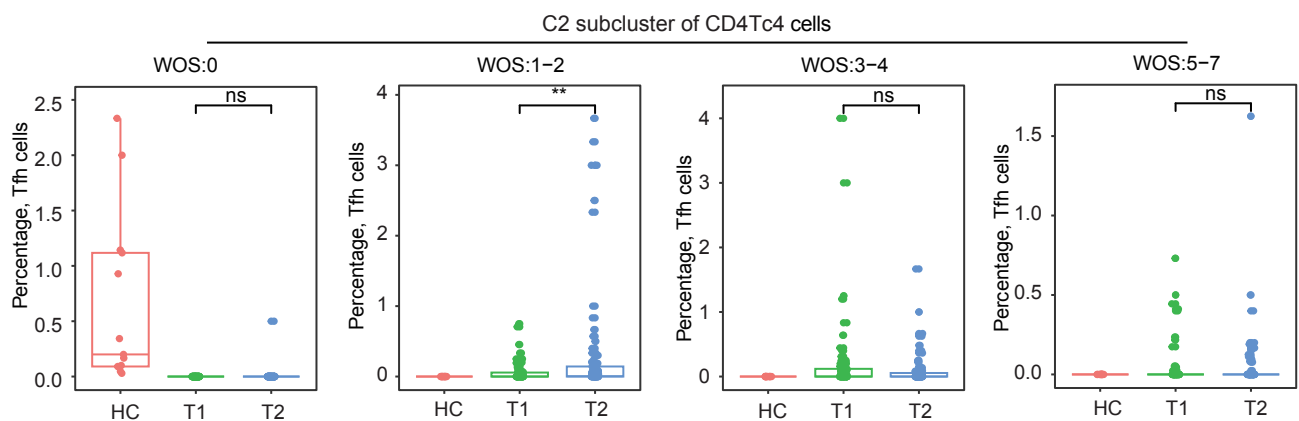

Supplement: qzaf019_Supplementary_Data [file qzaf019_supplementary_data.zip › Figure S7.pdf]

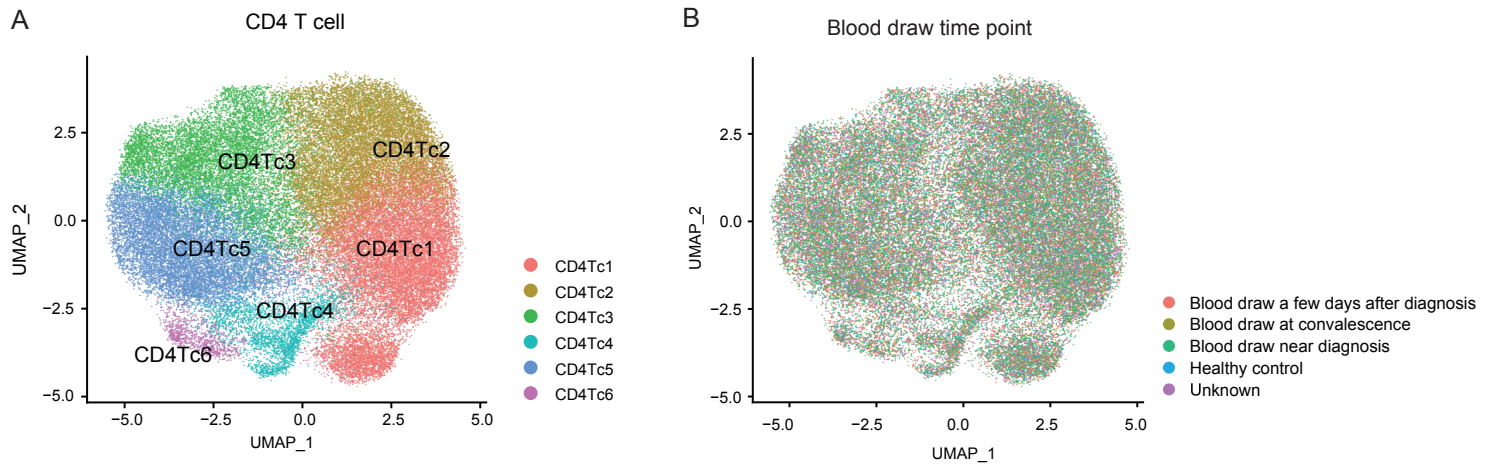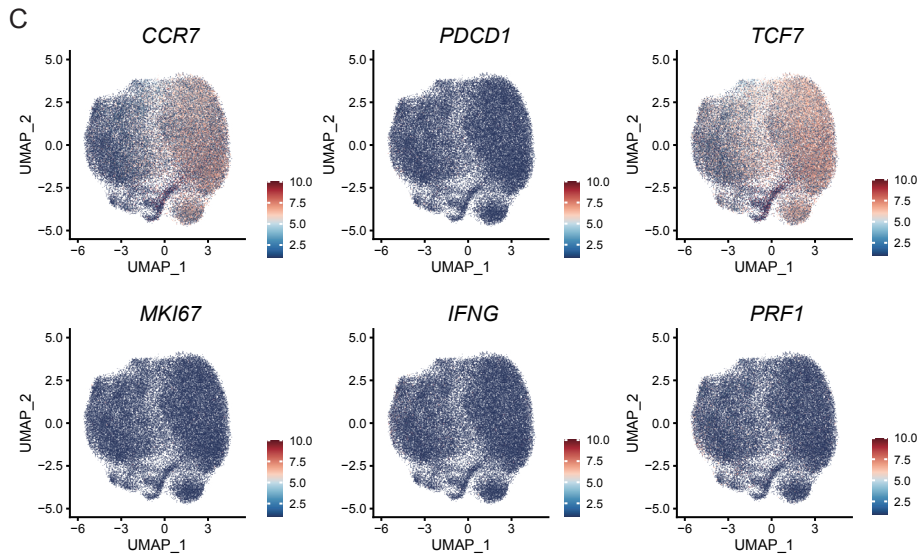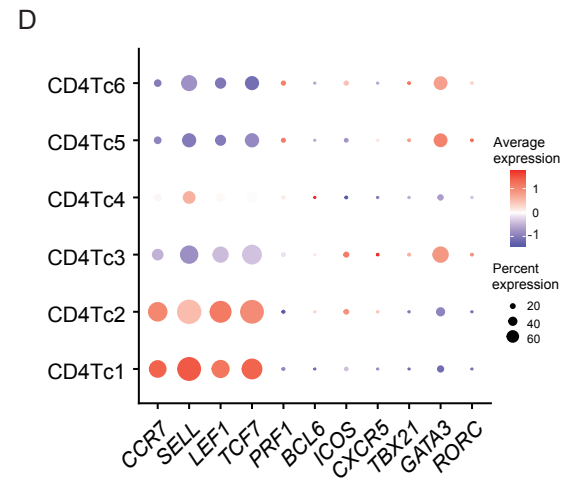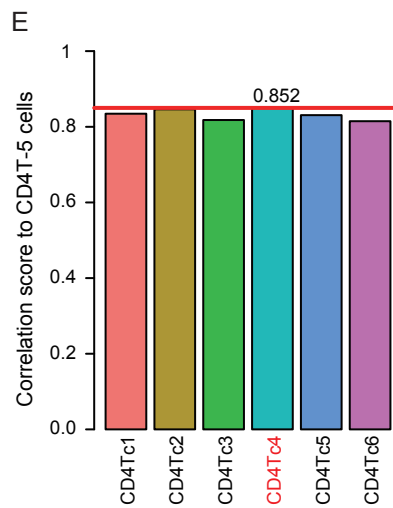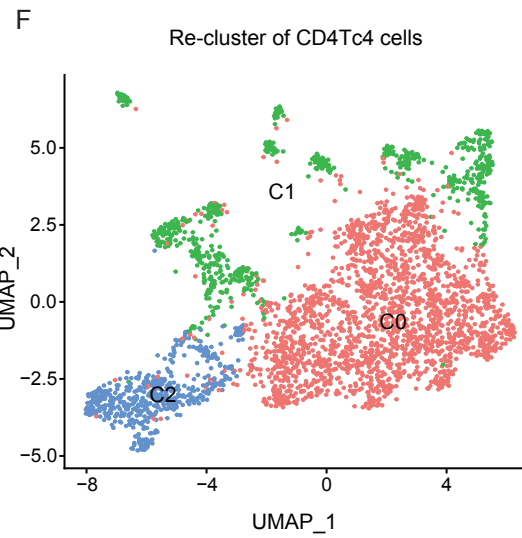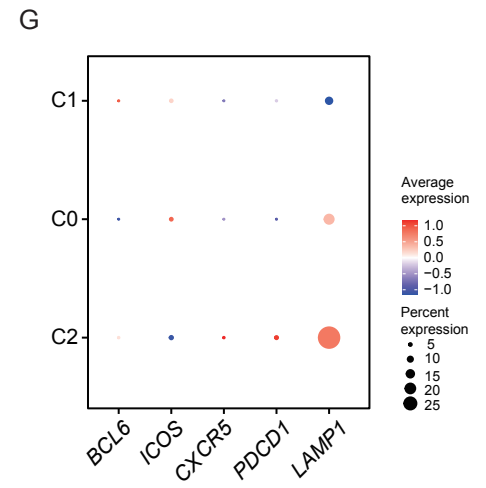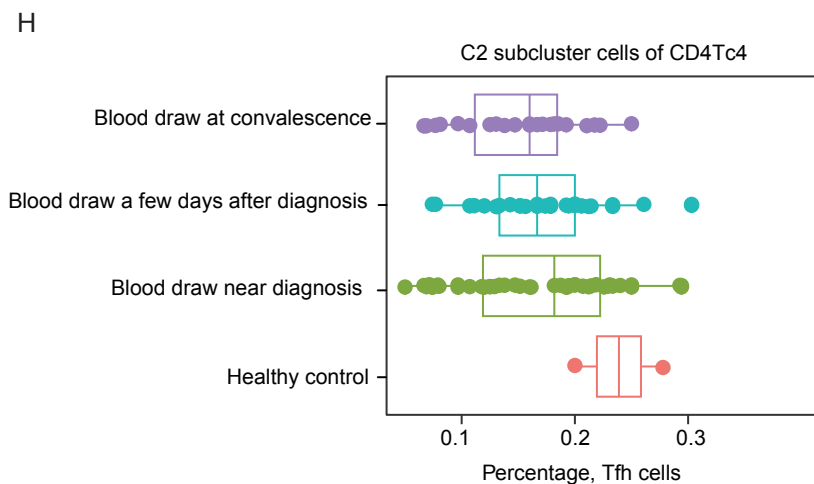

Supplement: qzaf019_Supplementary_Data [file qzaf019_supplementary_data.zip › Figure S8.pdf]

# aAPC-A02+SARS-CoV-2 peptides

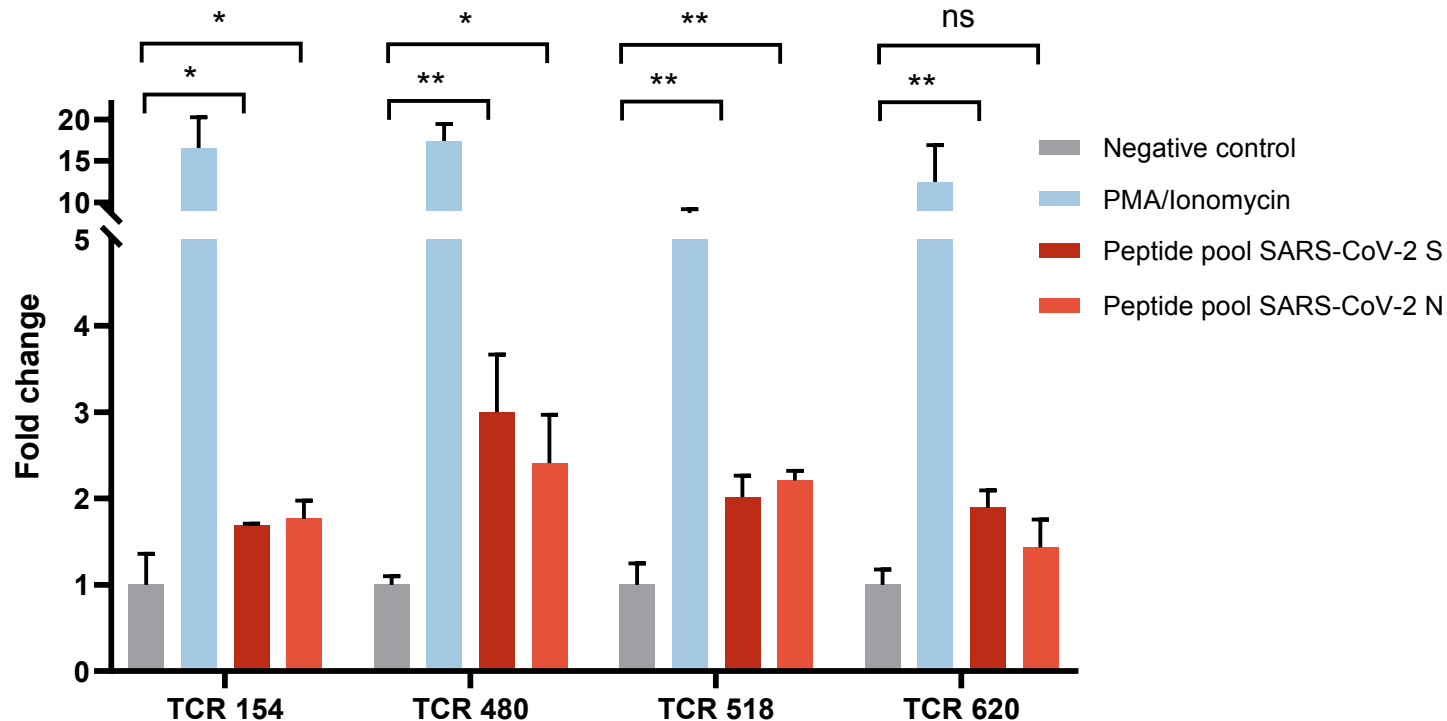

Supplement: qzaf019_Supplementary_Data [file qzaf019_supplementary_data.zip › Figure S9.pdf]
